# Synthesis, development, characterization and effectiveness of bovine pure platelet gel-collagen-polydioxanone bioactive graft on tendon healing

**DOI:** 10.1111/jcmm.12511

**Published:** 2015-02-20

**Authors:** Ali Moshiri, Ahmad Oryan, Abdolhamid Meimandi-Parizi

**Affiliations:** a Division of Surgery and Radiology, Department of Clinical Science, School of Veterinary Medicine, Shiraz UniversityShiraz, Iran; b Department of Pathology, School of Veterinary Medicine, Shiraz UniversityShiraz, Iran

**Keywords:** bovine platelet gel, tissue engineering, regenerative medicine, healing, modelling, remodelling, bioactive graft, growth factors

## Abstract

Bovine platelet gel (BPG) is an accessible and cost-effective source of growth factors which may have a value in tendon regenerative medicine. We produced a collagen implant (CI) as a tendon proper, covered it with polydioxanone (PDS) sheath to simulate paratenon and finally embedded the BPG as an active source of growth factor within the bioimplant to test whether BPG would be able to accelerate and enhance tendon regeneration and repair. After *in vitro* characterization of the bioactive grafts, the grafts were implanted in rabbit large tendon defect model. Untreated tendons and tendons treated with either CI or CI-PDS were served as controls for the CI-PDS-BPG. The animals were investigated clinically, ultrasonographically and haematologically for 120 days. After euthanasia, dry matter content, water uptake and delivery characteristics and also gross morphological, histopathological and scanning electron microscopic features of the healing tendons were assessed. *In vitro*, the activated platelets in the scaffold, released their growth factors significantly more than the controls. BPG also increased cell viability, and enhanced cellular differentiation, maturation and proliferation inside the CI-PDS compared with the controls. *In vivo*, the BPG modulated inflammation, increased quality and rate of fibroplasia and produced a remodelled tendon that had significantly higher collagen content and superior collagen fibril and fibre differentiation than controls. Treatment also significantly improved tendon water uptake and delivery characteristics, animals’ serum PDGF level, CI-PDS biocompatibility and biodegradability and reduced peritendinous adhesions, muscle fibrosis and atrophy. BPG was effective on tendon healing and CI-PDS-BPG may be a valuable bioscaffold in tendon reconstructive surgery.

## Introduction

Large Achilles tendon defects could occur because of tumours, severe traumas, infective and gangrenous ulcers, deep wounds, tendinopathies and several other reasons 1–4. In such cases, the diseased tissue should be removed and the defect should be reconstructed by auto- and or allografts 1,5. Using autografts has some significant limitations including donor site morbidity, pain and cosmetic concerns which are not clinically pleasant. In addition, a sufficient amount of autogenous tissue may not be available for reconstructing such large defects. Moreover, autograft procedure requires double surgery which is time consuming and increases the cost 5,6. In contrast to autografts, allografts have limitations, too. Allografts may be rejected by the host and have lower healing capability than autografts. In addition, both the fresh and processed allografts may transmit serious infection diseases such as hepatitis and human immune deficiency virus. Moreover, ethical concerns are other limitations of using allografts. Xenografts are not a popular option because they are acutely rejected by the host and may transfer many known and unknown zoonotic diseases to the patients 7. In addition to the difficulties of large tendon defect reconstruction, tendon healing has considerable limitations including development of peritendinous adhesions, poor healing response and the low quality of the repaired tissue which increases the chance of dehiscence. Therefore, revision surgery may be required increasing the cost 7.

Tissue engineering and regenerative medicine could be determined as an alternative option, but the efficacy of tissue engineering approaches is still under investigation 8,9. As a first step in tendon tissue engineering, a suitable scaffold should be designed and produced for defect reconstruction 10–12. Such scaffold should be biocompatible, biodegradable and biologically active, *in vivo*. However, recent investigations suggest that although tissue scaffolds may be useful for wound repair, they cannot provide all the necessary requirements for the healing process, solely 8,13. As a second step, the healing process and scaffold properties should further be enhanced by embedding the healing promotive factors and agents within tissue scaffolds 7,14,15.

The role of growth factors, glycosaminoglycans and several other agents has been investigated on tendon healing 4,13. Growth factors are responsible for many healing events after injury; however, they are expensive to be useful for clinical application 7,14,16. In the last decades, platelets have been introduced as a reliable, cost-effective and accessible source of growth factors 14,17,18. Upon activation, platelets release their growth factors at the wound site which trigger cellular migration, attachment, proliferation, differentiation and matrix production 4. Platelet-derived growth factors can also accelerate tendon healing by modulating the inflammation and accelerating the fibroplasia and remodelling 4,19,20. The most famous source of platelets that has been widely used in orthopaedic surgery and research is platelet-rich plasma (PRP) 3,21,22. PRP can be produced by one or two step centrifugation 21. Through the first centrifugation, RBCs that are harmful for the healing process because of their free radicals, are removed from the solution 23,24. Those PRPs that are produced after first centrifugation have higher WBC concentration which is beneficial for prevention of post-operative infections 24,25. However, WBCs may trigger exuberant inflammatory reaction at the wound site which is not clinically acceptable 26. Therefore, their concentration can be reduced by another centrifugation step 4.

Other forms such as platelet gel or platelet-fibrin glue have been shown to have higher efficacy in tendon healing because of their more biologically active nature than PRP 4,20,27. To date, autogenous form has been used in the clinical setting and autogenous and allogenous forms have been used in animal studies 19,28. In general, platelets have been shown to be more effective in animal studies when compared to the clinical studies 4. In addition, recent studies have tested the xenogenous form of platelets in animal models of bone healing and the results were encouraging 15,29,30. Bovine platelets are accessible and cost-effective source of platelets which may have a value in tissue engineering approaches.

Given the above explanations, we produced bovine platelet gel (BPG) embedded within a collagen implant (CI) as a tendon proper and covered it with a bidimensional polydioxanone (PDS) sheath to simulate paratenon. *In vitro*, we tried to characterize and find out the mechanism of BPG on the behaviour of the seeded fibroblasts on the scaffolds. *In vivo*, the role of CI-PDS-BPG on large Achilles tendon defect model was investigated in rabbits. Our hypotheses were that the BPG and its growth factors may regulate inflammation at short-term, trigger fibroplasia and remodelling at mid to long-term respectively. It is possible that bovine platelets because of its growth factors enhance cellular migration, proliferation and differentiation, and also they may improve the graft behaviour such as biocompatibility and biodegradability, *in vivo*. We tested our hypothesis by various basic to clinical methods.

## Materials and methods

### Production of collagen implant and polydioxanone sheath

Collagen type I was extracted from the bovine tendon and its purity was confirmed by SDS/PAGE 31. The acid-solubilized collagen molecules were electrospinned onto a dual plate device to produce the large and aligned electrospun collagen fibres 31. After electrospinning, the acid-solubilized bovine tendon type I collagen molecules were mixed with electrospun collagen fibres and polymerized in an incubator at 4°C for 48 hrs to produce a tridimensional collagen gel 8. The collagens were aligned under 12 Tesla magnetic fields (CRETA, Grenoble) during polymerization 13. The collagen composite was cut into several pieces of the same size and shape as the rabbit's Achilles apparatus (L, 20 mm; H, 3.5 mm; W, 3 mm). The collagen composites were cross-linked after suspension in iso-osmolar 0.1% riboflavin solution, using UV (wavelength of 365 nm) irradiation to increase the mechanical properties of the scaffold enabling it to hold suture tensions 8. Polydioxanone sheet (50 mm × 50 mm) was purchased (Johnson & Johnson, San Diego, CA, USA), cut and sectioned in smaller pieces (H, 20 mm; L, 14 mm; Thickness, 100 μm), melted at 40°C, and wrapped around each collagen piece (dimensions: 20 × 3.5 × 3 mm) to produce a CI-PDS sheath 8. The implants were left to dry at room temperature to remove the chemical solvents to prevent *in vivo* tissue reaction. The final product was repeatedly washed with distilled water, and received 100 Gray g-radiation and suspended in ethanol 96% to produce and maintain its sterility until surgery 13.

### Production of platelet gel embedded the artificial tendon

**Figure 1:**
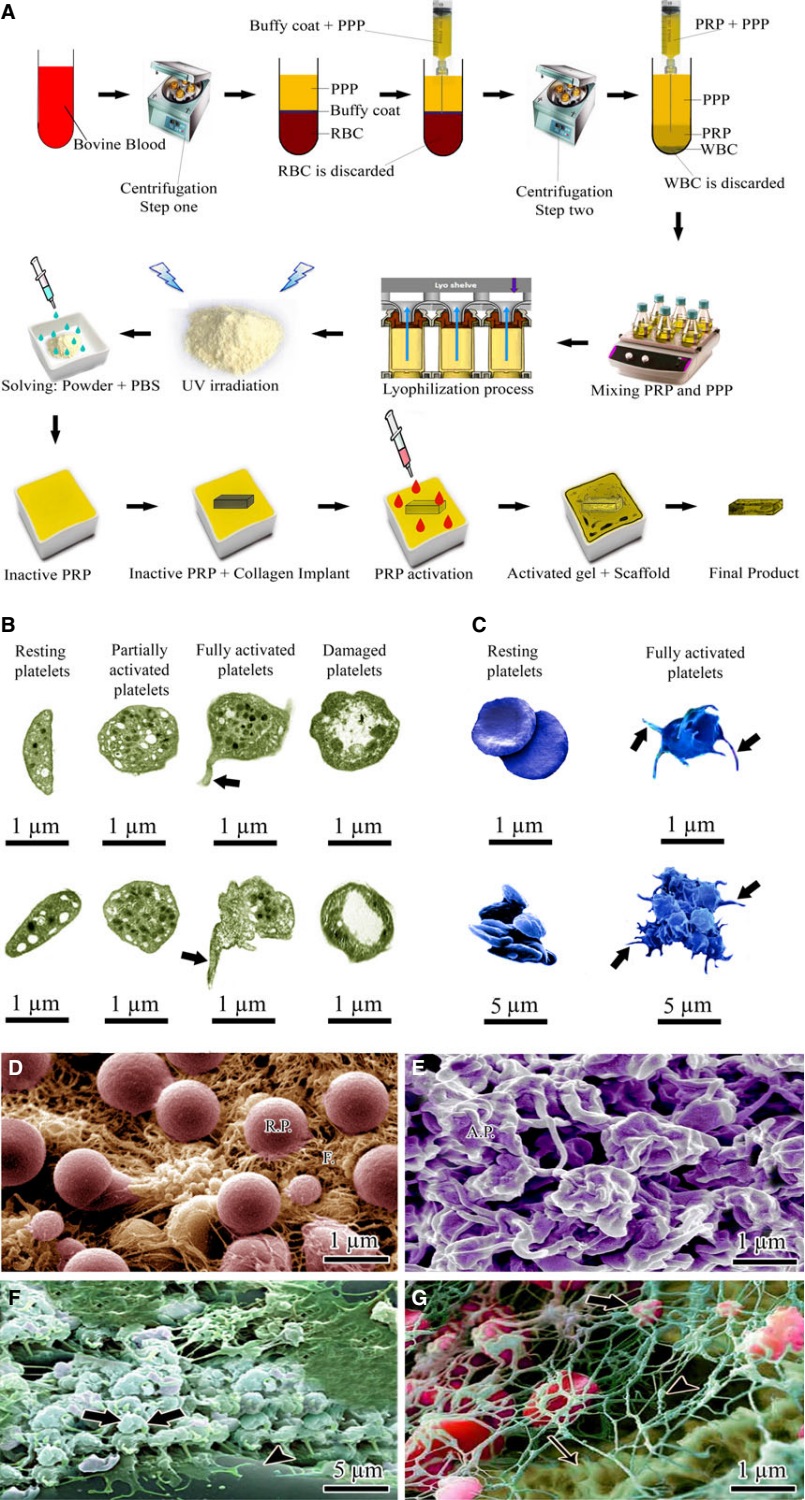
Development of platelet gel embedded within the artificial tendon and the platelets morphology. (A) Schematic view of the xenogenous-based BPG preparation and its embedding within the implant: Peripheral blood was obtained through IV catheterization of healthy bovines, and was transferred to EDTA tubes to prevent blood coagulation. The samples were centrifuged twice. After the first centrifugation, three layers were formed, including: (1) platelet-poor plasma (PPP), (2) Platelet-rich plasma (PRP) + white blood cells = buffy coat, and (3) red blood cells. The PPP and buffy coat layers were suctioned and transferred into a new tube. The RBCs were discarded. The PPP and buffy coat were again centrifuged. This resulted in the formation of another three layers, including: (1) PPP, (2) PRP and (3) WBC. WBCs were discarded, and the PPP and PRP were suctioned into the sterile tubes. The solution was mixed and lyophilized to concentrate the platelets and produce a lyophilized platelet powder. The powder was then sterilized through UV irradiation, and it was then solubilized in sterile phosphate buffer saline to produce the desired concentration (six to seven times greater than the physiological blood concentration). The sterile fully dehydrated three-dimensional collagen implant was immersed in the PRP solution, and the implant was left to absorb the platelets in its architecture. The PRP solution was then activated, using bovine thrombin and CaCl_2_. Therefore, the absorbed PRP in the implant was activated and the gel was formed within it. The final product was a platelet gel embedded within the implant which was then wrapped with PDS sheath. Each arrow shows a subsequent step. (B) Transmission electron microscopic images of bovine platelets in different statuses. (C) Scanning electron microscopic (SEM) views of resting and activated platelets. Resting platelets have an oval shape, smooth edges and randomly dispersed granules (B and C). Fully activated platelets have irregular shape, emission of pseudopods (thick arrows) and centralization of granules (B and C). Irreversibly damaged platelets have discoidal shape with the migration of organelles to the periphery (B). (D) SEM image of resting platelets (R.P.) within the PRP solution. (E) SEM image of fully activated platelets (A. P.) in the BPG. (F and G) SEM images of the A. P. (thick arrows) inside the collagen implant. Loose fibrin matrix has been indicated by arrow head and collagen fibres have been indicated by thin arrow.

Peripheral blood was harvested from healthy bovines and transferred into the sterile ethylenediaminetetraacetic acid (EDTA) tubes (1.5 mg/ml blood). We collected the blood in EDTA because based on our observations, the bovine platelets had less tendency for aggregation in EDTA blood samples than those of the acid citrate dextrose or citrate phosphate dextrose blood samples. The animals were free of any infective and zoonotic diseases such as bovine spongiform encephalitis, rabies, tuberculosis and brucellosis, and the safety of the blood samples were tested and approved by a certified laboratory (Masoud Lab, Tehran, Iran). On centrifugation of anti-coagulated blood at 215 g force (1500 R.P.M.) for 15 min., the following three layers were formed: red blood cells (bottom); white blood cells/platelets (buffy coat) (middle) and plasma (top) 21,32. The plasma and buffy coat layers were suctioned into new tubes and centrifuged again. Three layers including WBCs (bottom), PRP (middle) and platelet-poor plasma (PPP; top) were formed. Under stereomicroscope, the PPP and PRP were suctioned into new tubes. Three samples were fixed on glass slides and stained with Wright–Giemsa staining; it was confirmed that the samples were free of bovine WBCs and RBCs. We removed the RBCs because our initial tests showed that presence of the RBCs in the platelet solution increases the tendency of the platelets for aggregation. Because our PRP was xenogenous-based, harvested from bovine, we tried to decrease the immunogenicity of the PRP by removing the WBCs from the solution. The platelets were counted with a standard haemocytometer, and the total platelet count was calculated for each sample. After PRP + PPP preparation, the samples were lyophilized and pulverized. The powder was sterilized *via* UV irradiation and the sterile powder was solved in the sterile PBS (0.9% NaCl). The final platelet concentration per each μl was set to be 2,000,000 platelet (six to seven times greater than the physiological concentration) 21. Two millilitre of PRP was transferred to a sterile costume-made rectangular dish. The fully dehydrated CI-PDSs were weighed and then placed in the dish. After 30 min., the scaffolds fully absorbed the solution. Two millilitre of platelets were activated using a combination of bovine thrombin (5000 unit) and 5 ml of CaCl_2_ 10% with a proportion of 10 platelet solution:1 activator. This produced a BPG embedded within the CI-PDS. The hybrid scaffolds were then air-dried and placed in a sterile package for further use. The presence of the platelets and their attachment to the collagen fibres of the implant were confirmed by SEM, TEM and light microscopy (Fig.1).

### *In vitro* evaluations

The morphology of the scaffolds was studied by SEM and light microscopy 31. Three groups of scaffolds (each had 10 samples) were investigated including CI, CI-PDS and CI-PDS-BPG. For light microscopic studies, the scaffolds were fixed in buffered formalin 10%, and routine preparation method was employed to produce the longitudinal and transverse sections (4-μm thickness). For SEM analyses, the scaffolds were fixed in cold glutaraldehyde 2.5%, dehydrated in a graded series of ethanol, dried at room temperature and finally gold coated. Orientation of the collagen fibres and homogenous distribution of the platelets over and inside the scaffolds were evaluated. In addition, the platelets were counted in 10 different parts of each scaffold. Sterility and endotoxin content were tested *in vitro* and confirmed by microbiological and Limulus amebocyte lysate test respectively 8.

#### Coagulation profile – activated partial thromboplastin time and prothrombin time

The coagulation profile of the normal blood, platelets produced after first step centrifugation (PFSC), platelets produced after second step centrifugation (PSSC) and platelets produced after lyophilization and saline solving procedure (PLSSP) were measured, using STA Compact MAX instrument (Stago Diagnostica, Paris, France) according to the method described by Ng *et al*. 33.

#### Degradation rate

The normal blood clot (NBC), PFSC, PSSC and PLSSP gel, were incubated at 37°C and observed periodically and finally graded after 30 days. Grading was 0 for no changes, 1 for 1–50% degraded and 2 for 50–100% degraded.

#### Level of platelet growth factor

The PDGF-AA, -AB and -BB and also the IGF-I levels of the PFSC (750,000 platelets/μl, *n* = 10), PSSC (1,500,000 platelets/μl, *n* = 10), the PLSSP (2,000,000 platelets/μl, *n* = 10) and the PG (2,000,000 platelets/μl, *n* = 10) were measured, using a commercially available Quantikine ELISA kit (DHD00, DG100 respectively, R&D Systems, Minneapolis, MN, USA). To measure the PDGF-AA, -AB and -BB levels, the samples and standards were prepared according to the manufacturer's protocol. Briefly, the samples were incubated for 2 hrs, washed and incubated with enzyme conjugated antibodies directed against PDGF-AA, -AB and -BB for an additional 2 hrs at RT. The wells were then washed and the substrate was added and left for 20 min. at RT. The stop solution was added to each well and the absorbance was determined at 450 nm, using a microtitre plate reader. For measuring IGF-I, a dilution series of IGF standards was prepared in 100 μl volumes in 96-well microtitre plates coated with a monoclonal antibody specific for IGF-I. The microtitre plate was incubated for 2 hrs at 2–8°C. The wells were washed three times and incubated with enzyme conjugated IGF-I for 1 hr at 2–8°C. The wells were washed three times again, the substrate solution was added, and the plates were incubated for 30 min. at RT. The stop solution was added to each well, and absorbance was determined, at 450 nm, using a microtitre plate reader 34.

#### Platelet aggregation test

Determining the functional activity of the platelets is important during preparation of different platelet-based products such as PRP and platelet gel. The functionality of the platelets may be altered during different processizations. Light transmission aggregometry (LTA) is the most common method used to assess platelet function. LTA measures the changes in transmission of a beam of light through a sample of PRP or platelet suspensions in buffer, which occur when platelets change shape and aggregate upon stimulation. In the LTA method (Chrono-log series 400; Harvertown, PA, USA), collagen (3.2 μg/ml) and TRAP-6 (32 μM; Verum Diagnostica, München, DE, Germany) were used as agonists. The results were expressed as maximal light transmission. The LTA of the PFSC (750,000 platelets/μl, *n* = 10), PSSC (1,500,000 platelets/μl, *n* = 10) and PLSSP (2,000,000 platelets/μl, *n* = 10) were statistically compared 35.

#### Live/dead cell assay and immunofluorescence microscopy

Rat skin fibroblasts (cell line CRL-1213) were obtained from the American Type Culture Collection (Manassas, VA, USA) and cultured (37°C, CO_2_ 5%, pH = 7.4) in DMEM supplemented with 10% foetal bovine serum, 20 U/ml penicillin and 20 μg/ml streptomycin (Invitrogen, Carlsbad, CA, USA). Cell culture medium was replaced every 3 days. The cells were passaged at confluence and the 4–8th passage fibroblasts were used for the seeding. A conventional static seeding method was used to seed cells onto the CI, CI-PDS and CI-PDS-BPG scaffolds. Briefly, 50-μl cell suspension containing about 5 × 10^5^ cells was cultivated on every CI, CI-PDS and CI-PDS-BPG scaffolds and incubated for 1.5 hrs, allowing the cells to attach to the implants under standard culture conditions (37°C, in a humidified atmosphere containing 5% CO_2_ and 95% air). The remaining 950-μl media was then added to the wells. After 24 hrs, the non-adhering cells were washed off with PBS for three times and the samples were transferred to new tissue culture plates. The cell-seeded scaffolds experienced a 20-day static culture. In the culture period, the media were first replenished after the first 7 days and then every 3 days. During the culture period, cell viability was determined by live/dead cell assay using fluorescein diacetate (FDA, Molecular Probes, Invitrogen Corporation) (live) and propidium iodide (Cayman Chemical Company, Ann Arbor, MI, USA) (dead) on days 5, 10 and 20 after cell seeding and culture. Briefly, the cultured scaffolds were rinsed with PBS to remove non-adhering cells and were incubated in appropriate amounts of fluorescent dye for 45 min. at 37°C. The scaffolds with the fluorescence stained cells were viewed under a fluorescent microscope (Nikon Inc. Melville, NY, USA). Cells (*n* = 5 × 10^5^) cultured in the 3D culture medium were served as negative control for the CI, CI-PDS and CI-PDS-BPG. The viability index was analysed as: number of viable cells/total number of cells × 100. In each (*n* = 10 for each) groups of scaffolds, (3D culture system *versus* CI *versus* CI-PDS *versus* CI-PDS-BPG) 10 photomicrographs (magnification = ×200) were used for counting the viable and non-viable cells provided from 10 different areas of the scaffold (totally 100 photomicrograph for each scaffold). Cell–scaffold interaction was observed under SEM.

Cell morphology and cell–scaffold interaction were also studied by immunofluorescence microscopy. The scaffolds with cells on a 25-mm coverslip were washed twice with PBS. The cells were fixed in 4% paraformaldehyde, at RT, for 60 min. The fixed cells were then washed twice with 0.02% PBS/sodium azide and permeabilized with 0.2% saponin for 10 min. The non-specific sites were blocked by incubation in 0.02% PBS/1% BSA/0.02% sodium azide for 10 min. at RT. The primary antibody (anti-Grp78, also known as BiP at a 1:100 dilution) was added to the coverslip to completely cover the surface and allowed to incubate at RT for 45 min. The coverslip was rinsed three times with 0.02% PBS/sodium azide, and then blocked again with PBS/BSA/sodium azide for 10 min. The secondary antibody (Alexa 488 goat anti-rabbit at a 1:100 dilution) was added to the coverslip and incubated at RT for 45 min. The coverslip was rinsed three times with PBS/sodium azide, and then incubated in 2 ml of a DAPI (4′,6-diamidino-2-phenylindole) solution of 0.5 ng/ml for 15 min. The coverslip was rinsed with PBS/sodium azide, then with deionized water and mounted on a glass slide with Fluoromount G. To study the production of collagen type I in the cultured fibroblasts on the scaffolds, monoclonal antibody for type I collagen (1:20 dilution) obtained from EMD Chemicals (Calbiochem, San Diego, CA, USA) and secondary antibody Gt × Rb IgG Fluor (Chemicon, Temecula, CA, USA; 1:200 dilution) were used. The nucleus was also stained with DAPI.

### *In vivo* experiment

#### Study design

A number of 160 white New Zealand male rabbits (12–14-month age) were randomly assigned to control (defect only), CI, CI-PDS and CI-PDS-BPG (each had 40 rabbits). In each group, the animals were evaluated at 60 (*n* = 20) and 120 (*n* = 20) days after tendon injury (DAI) and surgical reconstruction. Another 40 rabbits were used as pilot groups and were randomly assigned to four groups the same as experimental animals (each had 10 rabbits). In each group, the animals were evaluated at 20 (*n* = 5) and 40 (*n* = 5) DAI to study the host–implant interactions. The animals had free access to food and water and maintained individually in standard rabbit cages during the experiment.

#### Tendon injury and surgical reconstruction

The animals were premedicated by intra-muscular (IM) injection of 1 mg/kg Acepromazine maleate and were anaesthetized by IM injection of 60 mg/kg Ketamine + 0.1 mg/kg Xylazine hydrochloride (All from Alfasan Co, Woerden, the Netherlands) 8. Under aseptic condition, a longitudinal skin incision was made over the Achilles tendon complex, then, 2 cm of the Achilles tendon with the covering paratenon was completely excised by transverse incisions. Primary reconstruction of the tendon was undertaken, using double-stranded modified Kessler core pattern, by monofilament absorbable PDS suture material (PDS 0-4, Ethicon, INC.1997, Johnson & Johnson). This aligned the remaining tendon extremities in a normal anatomical position, and produced a 2-cm gap between the tendon ends. The same method was applied to all groups by the same surgeon. For insertion of the implants in the tendon gap, a double-stranded suture was passed through the longitudinal axis of the implants. The subcutaneous tissue and skin over the lesion were closed in a routine fashion. Post-operative analgesia with fentanyl (Matrifen, Roskilde, Denmark; 0.0015 mg/kg/h) patch was provided for 3 days 13.

#### Pre-euthanasia assessments

Tarsal flexion degree, weight distribution per limb, pain on palpation, heel and toe position and swelling of the injured area were weekly monitored and scored (Table S1). Transverse diameter and surface temperature of the injured and contralateral part were weekly measured by digital calliper (Guanglu electronic digital caliper, Anyang, South Korea) and laser thermometer (Mastech, MS6530 Infrared Thermometer, Seoul, South Korea) respectively 8. Ultrasonography of the injured and contralateral tendons was performed weekly and the echogenicity, homogenicity, peritendinous adhesion, quality and volume of the regenerated tissues were assessed and scored (Table S2). Haematological samples were harvested from the ear vein of the animals at days 0, 10, 60 and 120 DAI and the percentage of neutrophils and lymphocytes, and number of the platelets, RBCs and WBCs were determined by cell counter (Veterinary Auto-analyzer, Cambridge, UK) 31. In addition, serum PDGF level of the animals was measured at days 60 and 120 DAI using commercial ELISA kit: platelet-derived growth factor-AA (PDGF-AA) and -AB Immunoassay (Biotrend Chemicals, LLC136 South Holiday Road, Unit C, Destin, FL, USA) 31.

#### Ethics and euthanasia

All animals received humane care in compliance with the Guide for Care and use of Laboratory Animals (NIH publication No. 85-23, revised 1985). The study was approved by the local Ethics Committee of our Veterinary School. The animals were anaesthetized by IM injection of 60 mg/kg Ketamine + 2 mg/kg Xylazine + 1 mg/kg Acepromazine maleate (All from Alfasan Co). Then, they were killed by intra-cardiac injection of 1 mg/kg Gallamine triethiodide (Specia Co., Paris, France). The mean duration of euthanasia was 5.57 sec. 8.

#### Post-euthanasia assessments

In each group and at each time-point, all the tendon samples harvested from the animals were evaluated at gross pathological level (*n* = 20 for experimental and *n* = 5 for pilot animals). At gross pathological level, hyperaemia, development of peritendinous adhesions, general appearance of the tendon proper, gastroc-soleus muscle atrophy and fibrosis together with the tendon diameter were scored and directly measured (Table S3). Then, tendon samples were longitudinally divided into three sections. Part A was used for histopathology, part B for SEM and part C for determination of water uptake, water delivery, dry matter content and hydroxyproline. In histopathology, the tendon samples were fixed in buffered formalin 10%, dehydrated in a graded series of ethanol, cleared in xylene, embedded in paraffin wax, sectioned at 4 μm, and finally stained with haematoxylin and eosin staining. Histological sections were studied under light microscopy (Olympus, Tokyo, Japan). The employing magnifications were in the ranges of ×40 to ×1000. In each group (*n* = 20 L; *n* = 20 R), three tissue sections were provided from different depth of each tendon sample and five photomicrographs were provided from five histopathological fields in each tissue section. Totally, 300 histopathological fields were studied for each group. Number and diameter of total cells, immature and mature fibroblasts, fibrocytes, neutrophils, macrophages, lymphocytes and blood vessels were counted and measured using software (Adobe Photoshop CS-5, CA, USA). Crimp pattern, alignment, perivascular oedema, tissue maturity and vascularity were scored (Table S4). Other tissue characteristics were qualitatively reported by three expert pathologists. For SEM, the samples were fixed in cold glutaraldehyde 2.5%, coated by hexa-methyl-disilazane (TAAB, Co., London, UK), dried under a vacuum at RT, and finally gold coated. Using scanning electron microscopy (SEM; Cambridge, London, UK), different magnifications from ×50 to ×100,000 were used to analyse the morphological and morphometrical characteristics of the implants and tissue samples. Cell number, cell density, diameter of the collagen fibrils, collagen fibres, collagen fibre bundles and collagen density were measured 31. Collagen fibrils’ alignment and their maturity together with crimp pattern were scored (Table S5).

Dry matter content was calculated as: dry weight/wet weight ×100 13. For determination of water uptake and delivery, wet weight and dry weight of the samples were measured, then the fully dehydrated and fully hydrated tendon samples were rinsed in saline 0.9% and exposed to air at RT to absorb or evaporate the hydration respectively. The weight gain and weight loss of the samples were measured time dependently to calculate the water uptake and delivery indices respectively. Index of water uptake = Wdry/time. The time refers to the point that the sample gained its maximum Wwet. Index of water delivery = Wwet/Wdry × 100/time. The time refers to the point that the samples gained their maximum Wdry 31. The hydroxyproline concentration was measured by spectrophotometry 8. Briefly, the samples were hydrolysed in 6 M HCl at 105°C for 14 hrs and the hydroxyproline was oxidized by chloramines T, and then by adding Ehrlich's reagent and incubating at 60°C, a chromophore was formed. To remove the interfering chromophores, the hydroxyproline product in alkaline media was extracted into toluene and then into acid phase. The absorbance of acid phase was read at 543 nm and the hydroxyproline content was calculated from the calibration curve based on the standard solutions run the same as the samples.

#### Inter-tester and intra-tester reliability

The investigators who undertook the measurements and analyses of the results were unaware of the experimental design and grouping details. Each evaluation and measurement was performed in triplicate and the mean of the measured values were reported. In addition, in each method of evaluation, three expert scientists reported their results. No significant differences were seen between the results of the individuals (*P* > 0.05).

### Statistical analyses

All the quantitative values were expressed as mean ± SD. The significant differences in the measured values between multiple groups at one time-point (*e.g*. 60 DAI) were tested using one-way anova with its subsequent Tukey's *post hoc* tests and between multiple comparisons at multiple time-points, repeated measures anova was used. All scored values were expressed as median (minimum–maximum). Kruskal–Wallis *H*-test was performed to analyse the scored values. A *P* < 0.05 was considered statistically significant 13.

## Results

### *In vitro* findings

#### Morphology of the platelets

The non-activated PFSCs, PSSCs and also the PLSSPs were biconvex discoid structures shaped like a lens, 2–3 μm in greatest diameter. The activated platelets observed in the platelet gel had pseudopodia emission as confirmed by TEM and SEM. Collecting the whole blood samples in the EDTA did not have any deleterious effect on platelets morphology and their activation. Number of platelets/mm^3^ observed in the CI-PDS-BPG, was 2 × 10^6^. At SEM, the platelets and fibrin were evenly distributed and infiltrated throughout the CI-PDS-BPG so that the implant absorbed the platelets, homogenously. The structure of the platelets was confirmed by SEM, TEM and light microscopy. No significant differences were seen between number of the platelets in 10 different areas of the implants (*P* > 0.05). In the CI-PDS-BPG scaffolds, the proportion of the activated platelets/total platelets was 89.61 ± 6.54%. Such a proportion suggested that the activation method was effective and most of the platelets were activated inside the scaffolds.

#### Coagulation profile

**Figure 2:**
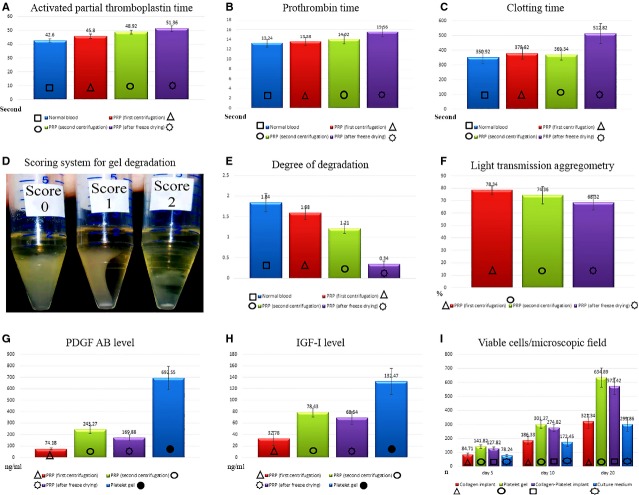
*In vitro* characteristics of the bovine platelet gel. We successfully developed a novel bovine platelet gel so that it had more function and quality than the PRP solution. Regarding activated partial prothrombin time (A) no significant differences were seen between different PRPs (*P* > 0.05). PRP produced after lyophilization and saline solving procedure (PLSSP) had significantly higher prothrombin (B) and clotting time (C) and lower degree of degradation (D and E) compared to the controls (*P* < 0.05). There were no significant differences between the light transmission aggregometry (F) of PLSSP and those produced after second step centrifugation (*P* > 0.05). Bovine platelet gel had significantly higher PDGF-AB (G) and also the IGF-I (H) level compared to the controls (*P* = 0.001 for all). The BPG and the CI-BPG had significantly higher number of viable cells (I) at various time-points compared to their controls (*P* = 0.001 for all).

Regarding the activated partial thromboplastin time, no significant differences were seen, between the NBCs and PFSCs, and also between the PFSCs and PSSCs (*P* > 0.05). However, lyophilizaion and the saline solving procedure (LSSP) significantly increased prothrombin and clotting time of the PLSSPs compared with the controls (*P* < 0.05; Fig.2).

#### Degradation rate

After the second step centrifugation, the degradation rate of the PRP significantly decreased compared with the controls (*P* = 0.001). LSSP significantly decreased the degradation rate of the BPG compared with the controls (*P* = 0.001; Fig.2).

#### Light transmission aggregometry

There were no significant differences between the LTA of PFSCs and PSSCs (*P* > 0.05). There were also no significant differences between the LTA of the PSSCs and those PLSSPs (*P* > 0.05; Fig.2).

#### Level of the platelet growth factors

After the second step centrifugation, the PDGF-AA, -AB and -BB and also the IGF-I level of the PSSCs were significantly higher than the PFSCs (*P* = 0.001). After LSSP, the PDGF-AA, -AB and -BB and also the IGF-I level of the PLSSPs significantly reduced when compared to the PSSCs (*P* = 0.001). Activation of the PLSSPs to platelet gel significantly increased the PDGF-AA, -AB and -BB and also the IGF-I level of the samples when compared to the controls (*P* = 0.001; Fig.2).

#### Cell viability, distribution and proliferation

**Figure 3:**
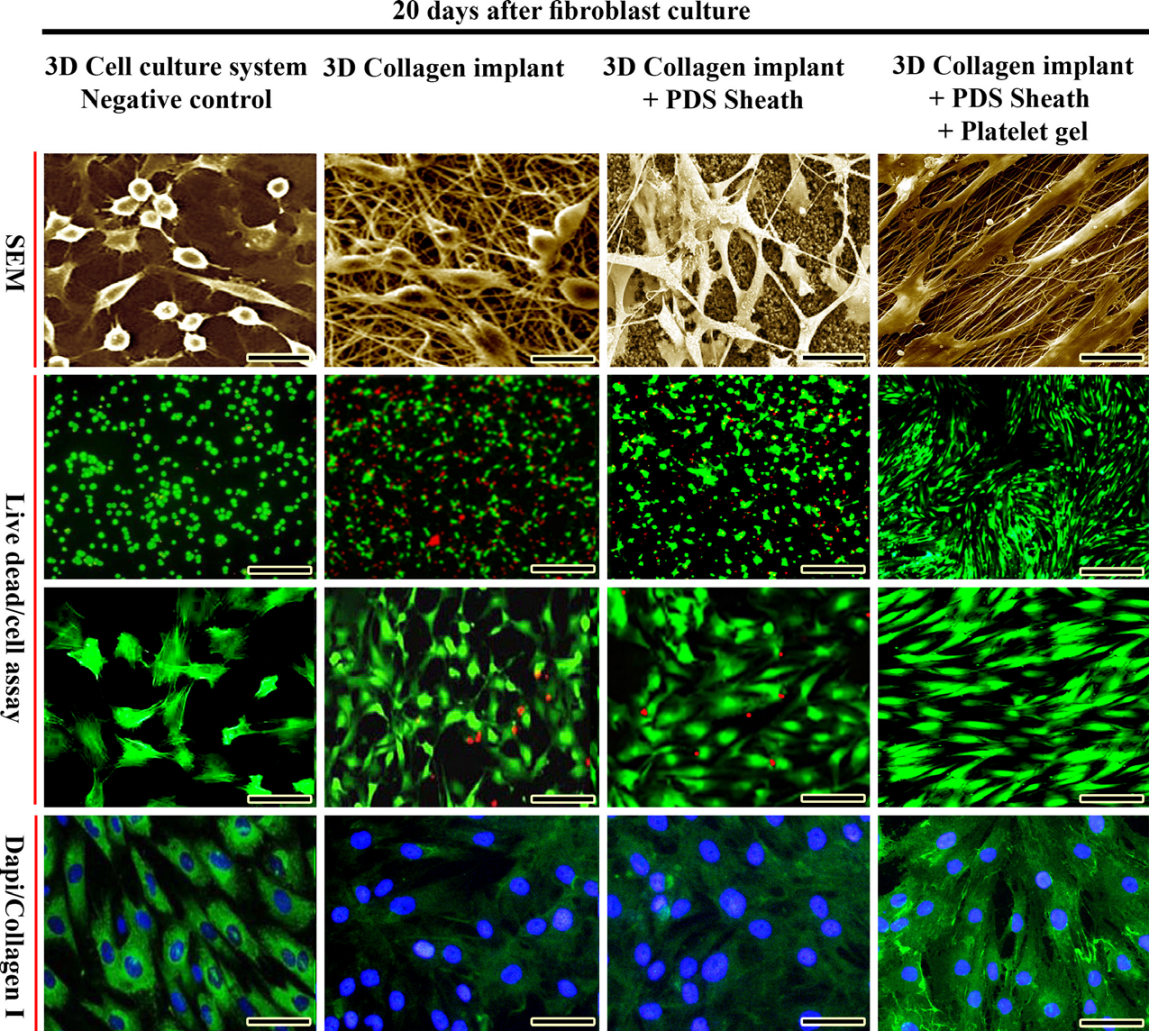
*In vitro* characterization of the bioactive graft. Note that bovine platelets improved cellular proliferation and distribution over the implant as it was shown by SEM images. The live cells have been stained by fluorescein dye acetate and dead cells have been stained with red propidium iodide. Note that the platelets increased the cell viability of the implants so that significantly higher viable cells could be seen in the CI-PDS-BPG group compared to the controls (*P* < 0.05). Fluorescence microscopy images of fixed rat fibroblasts on various implants, with monoclonal antibody for type I collagen conjugated with Gt × Rb IgG Flour (green)/DAPI (blue)-stained cells suggest that the bovine platelet gel when embedded within the artificial tendon increases the cellular proliferation, cell density and matrix production (*P* < 0.05). Note that the cultured cells on the platelet gel-artificial tendon produced more collagen type I than those cells cultured on the artificial tendon alone. Scale bar for SEM = 12 μm, for live dead cell assay = 50, 25 μm respectively, and for immune-fluorescence micrographs (DAPI-Collagen I) = 20 μm.

From 5 to 20 days after cell seeding and culture, almost all the fibroblasts in the CI-PDS-BPG were visualized as green, indicating that the cells were alive. Lack of propidium iodide (red)-stained dead cells suggests that normal rat fibroblasts were attached to the scaffold and that the majority of the cells were viable. BPG significantly increased the number of the viable cells in the CI-PDS-BPG compared to the CI and CI-PDS (*P* = 0.001). In addition, the BPG improved cellular proliferation, their maturation and matrix production so that most of the cells were laid in an aligned manner through one direction and their transverse and longitudinal diameters were shorter and larger than those seen in the controls, correspondingly (Figs2 and 3).

After 5, 10 and 20 days of fibroblast seeding and culture into the CI, CI-PDS and CI-PDS-BPG, the qualitative results of the SEM and immunofluorescence microscopic studies showed that BPGs resulted in a superior distribution of the cultured fibroblasts inside the scaffold and the cells were more mature and produced more collagenous matrix in the scaffold. In addition, the CI-PDS-BPG scaffolds had significantly higher cultured fibroblasts (counted at magnification of ×200) on day 5 (78.43 ± 12.76^CI^ *versus* 94.31 ± 15.35^CI-PDS^ *versus* 151.33 ± 22.49^CI-PDS-BPG^), 10 (157.28 ± 29.65^CI^ *versus* 167.34 ±21.53^CI-PDS^ *versus* 269.35 ± 43.19^CI-PDS-BPG^) and 20 (231.5 ±46.71^CI^ *versus* 252 ± 39.01^CI-PDS^ *versus* 486 ± 64.28^CI-PDS-BPG^) compared to the CI and CI-PDS (*P* = 0.001 for both).

In the CI-PDS-BPG, the cultured fibroblasts proliferated all over the scaffold both on the surface and also in the internal architecture of the scaffolds, so that the fibroblasts oriented in an aligned manner along the direction of the scaffold collagen fibres. There were no significant differences between number of the cultured fibroblasts in different parts of the CI-PDS-BPG scaffolds as confirmed by immunofluorescence microscopy, SEM and light microscopy (*P* > 0.05; Fig.1). In contrast to the embedded platelet gel scaffolds, although the fibroblasts proliferated in an align manner outside and inside the CI and CI-PDS scaffolds, number of the cultured fibroblasts were significantly higher in the peripheral parts than the inner parts of the scaffolds (*P* < 0.05). These results confirmed that the BPG triggered and accelerated the fibroblasts proliferation more homogenously and continuously all over the scaffolds.

### *In vivo* experiment

#### Pre-euthanasia assessments

##### Clinical findings

For translational purpose, the clinical examinations were done for the animals. None of the animals died during the course of the experiment and all had normal appetite and weight gain. Clinical scoring was performed weekly by three individuals who were expert doctor of veterinary medicine. Treatment with BPG, significantly improved tarsal flexion degree (*P* = 0.001, *P* = 0.001, *P* = 0.026), weight distrib-ution (*P* = 0.001, *P* = 0.001, *P* = 0.042), heel and toe posit-ion (*P* = 0.001, *P* = 0.001, *P* = 0.048), pain on palpation (*P* = 0.001, *P* = 0.001, *P* = 0.012) and swelling of the injured area (*P* = 0.001, *P* = 0.001, *P* = 0.058) compared to those that were left untreated or treated with either the CI or CI-PDS respectively (Table1).

**Table 1:** Clinical, ultrasonographical, gross morphological, histopathological and ultrastructural scoring values

Type of assessment	Scoring criteria	Control (1) Median (min–max)	CI (2) Median (min–max)	CI-PDS (3) Median (min–max)	CI-PDS-PG (4) Median (min–max)	*P*-value 1 *versus* 4	*P*-value 2 *versus* 4	*P*-value 3 *versus* 4
		*n* = 20	*n* = 20	*n* = 20	*n* = 20	*n* = 20	*n* = 20	*n* = 20
Clinical scored values (Sum scores of 17 weeks)	Tarsal flexion degree	50.5 (40–59)	39 (31–44)	36 (29–43)	26.5 (21–34)	0.001	0.001	0.026
	Weight distribution	56.5 (46–60)	45.5 (35–57)	36 (29–44)	24 (18–31)	0.001	0.001	0.042
	Pain on palpation	45 (39–48)	37 (32–41)	36 (32–40)	25 (20–34)	0.001	0.001	0.012
	Heel and toe position	46 (38–48)	34.5 (31–38)	31.5 (24–35)	23 (18–33)	0.001	0.001	0.048
	Swelling	47 (44–48)	40 (36–45)	36 (31–40)	30 (26–34)	0.001	0.001	0.058
Ultrasonographical scored values	Echogenicity	3 (2–3)	2 (1–3)	2 (1–3)	0.5 (0–2)	0.001	0.001	0.012
	Homogeneity	3 (3–3)	1.5 (1–2)	1 (1–2)	0 (0–1)	0.001	0.001	0.001
	Peritendinous adhesion	3 (3–3)	2 (1–3)	1 (0–2)	1 (0–1)	0.001	0.001	0.046
	Regeneration volume	5 (4–5)	3 (2–3)	2 (2–3)	0.5 (0–1)	0.001	0.001	0.001
	Intensity of the peritendinous adhesion	5 (5–5)	2.5 (2–4)	1 (1–2)	1 (0–1)	0.001	0.001	0.019
	Regenerative proportion	5 (4–5)	2 (2–3)	2 (1–3)	1 (0–2)	0.001	0.001	0.048
Gross morphological scored values	Peritendinous adhesion	3 (3–3)	2 (1–3)	1 (0–2)	1 (0–1)	0.001	0.001	0.042
	Hyperaemia	3 (3–3)	1 (1–2)	1 (1–2)	0 (0–1)	0.001	0.001	0.002
	General appearance	4 (3–4)	2 (2–3)	1 (1–3)	0 (0–2)	0.001	0.001	0.026
	Muscle atrophy	4 (4–4)	2 (2–3)	1 (1–2)	0 (0–0)	0.001	0.001	0.001
	Muscle fibrosis	4 (3–4)	1 (1–2)	1 (1–2)	0.5 (0–1)	0.001	0.001	0.003
Histopathological scored values	Alignment	3 (3–3)	1 (1–2)	0.5 (0–1)	0 (0–0)	0.001	0.001	0.001
	Perivascular oedema	3 (2–3)	1 (1–2)	1 (1–2)	0 (0–1)	0.001	0.001	0.001
	Tissue maturity	4 (4–4)	2 (1–2)	1.5 (0–2)	0.5 (0–1)	0.001	0.001	0.017
	Crimp pattern	5 (5–5)	3 (2–3)	1.5 (1–3)	1 (0–1)	0.001	0.001	0.002
	Vascularity	5 (5–5)	2 (1–2)	2 (1–2)	0 (0–1)	0.001	0.001	0.001
SEM scored values	Alignment	4 (4–4)	2 (1–2)	1.5 (0–2)	0.5 (0–1)	0.001	0.001	0.033
	Maturity of the collagen fibrils	4 (3–4)	1 (1–3)	1 (0–2)	1 (0–1)	0.001	0.001	0.048
	Crimp pattern	4 (4–4)	2 (1–2)	2 (1–2)	1 (0–1)	0.001	0.001	0.002

All scored values were expressed as median (min–max). Kruskal–Wallis *H*-test was performed to analyse the scored values. A *P* < 0.05 was considered statistically significant. For more information regarding scoring criteria please see Supplementary file 1.

##### Transverse diameter of the injured area

**Figure 4:**
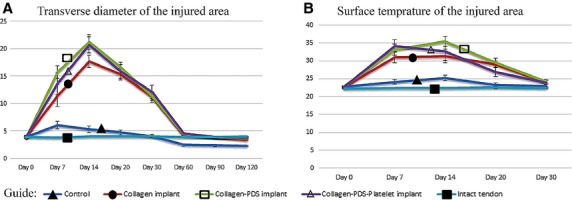
Transverse diameter and surface temperature of the injured area including skin, subcutaneous and the injured tendon. Note that bovine platelet gel embedded within artificial tendon (CI-PDS-BPG) significantly increased diameter of the injured area on day 14 after injury compared to the collagen implant (CI), defect and intact tendons (*P* < 0.05), but the diameter of the injured area of the BPG-treated tendon significantly decreased on days 20, 30 and 60 after injury compared to day 14 (*P* = 0.001 for all) (A). In addition, the CI-PDS-BPG significantly increased surface temperature of the injured area on day 7 after injury compared to the CI, defect and normal contralateral area (*P* < 0.05) but then it reduced surface temperature faster compared to the CI-PDS on day 20 after injury (*P* < 0.05) (B). Note that, no significant increase in transverse diameter (A) and surface temperature (B) is seen in the defect group compared to the treated groups suggesting the bioimplants triggered the inflammation during the first 2 weeks after injury.

There were no significant differences between the transverse diameter of the injured and contralateral tendons before and after the operation on day 0 (*P* < 0.05). After implantation of the prosthetic implants in tendon defect, transverse diameter of the injured tendons significantly increased on days 7 and 14 (*P* = 0.001) but then gradually decreased until day 120 after injury (*P* = 0.001). In the first 14 DAI, transverse diameter of the injured treated tendons with various implants was significantly higher than the injured control tendons (no implant; *P* = 0.001 for all). BPG did not increase the transverse diameter of the injured tendons compared to the collagen-PDS-treated tendons, but it slightly reduced it faster so that at the end of the experiment, the BPG-treated tendons had almost comparable transverse diameter to normal contralateral tendons (Fig.4).

##### Surface temperature of the injured area

There were no significant differences between the surface temperature of the injured and contralateral Achilles areas of different animals on day 0 and was in the range 22–23°C (*P* > 0.05). From day 7 to day 20 after tendon injury, those tendons treated by various implants had significantly higher surface temperature compared to the control (defect) group. BPG increased the surface temperature on day 7 but it decreased the surface temperature of the injured area faster when compared to the collagen and collagen-PDS-treated groups so that on day 20 after tendon injury, the platelet-treated group had significantly lower surface temperature compared to the collagen and collagen-PDS-treated groups (*P* > 0.05). Surface temperature of all the groups reached the normal values at 30 days after surgery (Fig.4).

##### Ultrasonography

**Figure 5:**
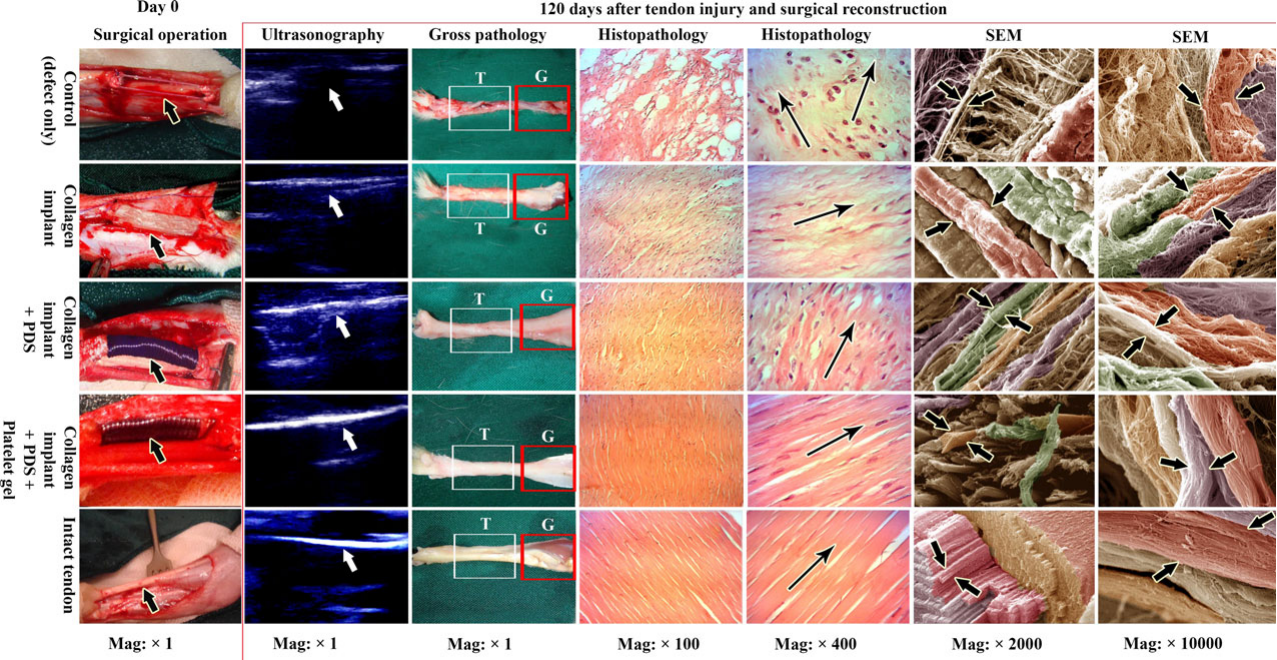
Morphological features of injured tendons after 120 days of tendon reconstruction. In surgical and ultrasonographic figures, the arrows show injured tendon. Note that the PBG-treated tendons have superior echogenicity and homogenicity compared to the controls (*P* < 0.05). In gross pathology, T: tendon; G: gastroc-soleus muscle. Note that treatment with BPG significantly reduced hyperaemia, peritendinous adhesions, muscle fibrosis and atrophy compared to the controls (*P* < 0.05). In histological figures, arrows show the direction of the collagenous tissue which has been regenerated in the defect area. Treatment with BPG significantly increased density of the collagenous tissue and reduced cellularity compared to the controls (*P* < 0.05). In SEM images, the arrows show collagen fibres. Note that treatment with BPG improved collagen fibril to fibre differentiation so that the newly formed collagen fibres are comparable to normal uninjured collagen fibres.

Treatment with BPG significantly improved the scoring values for the echogenicity (*P* = 0.001, *P* = 0.001, *P* = 0.012), homogeneity (*P* = 0.001, *P* = 0.001, *P* = 0.001), peritendinous adhesion (*P* = 0.001, *P* = 0.001, *P* = 0.046), regeneration volume (*P* =0.001, *P* = 0.001, *P* = 0.001), intensity of the peritendinous adhesion (*P* = 0.001, *P* = 0.001, *P* = 0.019), and regenerative proportion (*P* = 0.001, *P* = 0.001, *P* = 0.048) compared to the control, collagen and collagen-PDS-treated groups (Table1, Fig.5).

##### Haematology

No significant changes were seen for the number of RBCs between multiple groups at multiple time-points (*P* > 0.05). After implantation of various prosthetic implants (CI, CI-PDS and CI-PDS-BPG) in tendon defects, at 60 DAI, number of the leucocytes significantly decreased in all the treated groups compared to the control (defect) group but at that stage, those animals treated with BPGs had significantly higher number of leucocytes compared to those treated with CI or CI-PDS (*P* = 0.001). At 120 DAI, the leucocyte number significantly increased (*P* < 0.05) and reached its normal level in all the groups so that the statistics were not significant (*P* > 0.05). At 60 DAI, implantation of the CI and CI-PDS significantly reduced percentage of neutrophils compared to the control (defect) group (*P* = 0.001 for both). The BPG restored the percentage of neutrophil to normal value so that the platelet-treated animals had significantly higher percentage of neutrophils compared to those treated with either the CI or CI-PDS (*P* = 0.001 for both) at that stage. At 120 DAI, neutrophil percentage significantly increased (*P* < 0.05) compared to day 60 in all the groups and reached its normal level. Bovine platelets significantly reduced lymphocyte percentage in the injured animals compared to those treated with CI-PDS implants, at 60 DAI (*P* = 0.001). At 120 DAI, the lymphocyte percentage decreased to normal value in all the groups, but at this stage, the platelet-treated animals had still lower lymphocyte percentage compared to CI-PDS-treated animals. At 60 DAI, those animals treated with various prosthetic implants had significantly higher platelet count compared to the defect group (*P* = 0.001 for all). At 120 DAI, no significant differences were observed between the platelet counts in all the groups (Table2).

**Table 2:** Serum PDGF level and blood profile of the injured animals after 60 and 120 days of tendon injury and surgical operation

	60 days after tendon injury				120 days after tendon injury			
	Control (1)	CI (2)	CI-PDS (3)	CI-PDS-PG (4)	Control (5)	CI (6)	CI-PDS (7)	CI-PDS-PG (8)
PDGF-AA (ng/dl)	1.92 ± 0.93	8.23 ± 1.21	9.26 ± 1.35	17.24 ± 1.75	1.04 ± 0.51	3.08 ± 0.52	3.94 ± 0.47	7.57 ± 0.8
PDGF-AB (ng/dl)	5.32 ± 1.28	11.83 ± 0.58	13.63 ± 0.83	18.5 ± 1.63	2.91 ± 0.63	5.04 ± 0.68	5.75 ± 0.68	9.23 ± 0.88
Red blood cells (*n* × 10^6^/μl)	7.02 ± 0.68	6.85 ± 0.84	6.98 ± 0.82	6.72 ± 0.52	6.48 ± 0.84	6.41 ± 0.73	6.81 ± 0.84	6.47 ± 0.8
Leucocytes (*n*/μl)	8940.57 ± 720.04	5141.42 ± 755.64	4515.42 ± 721.1	5992 ± 607.72	8425.14 ± 336.68	8072.28 ± 681.48	7621 ± 589.01	8170.71 ± 671.57
Neutrophil (%)	57.71 ± 1.7	45.14 ± 1.95	43.57 ± 1.9	52 ± 1.29	66.85 ± 1.77	60.42 ± 2.22	58.71 ± 2.69	63.71 ± 1.49
Lymphocyte (%)	43 ± 1.34	49 ± 2	54 ± 1.73	49 ± 1.21	35 ± 2.26	37 ± 1.49	38 ± 2.58	35 ± 2.21
Platelet (*n*/μl)	153.57 ± 14.03	330.85 ± 26.49	355 ± 31.81	345.42 ± 41.66	338.28 ± 36.21	362 ± 27.21	345.85 ± 31.88	367.14 ± 16.25

All the quantitative values were expressed as mean ± SD. The significant differences in the measured values between multiple groups at one time-point (*e.g*. 60 DAI) were tested using one-way anova with subsequent Tukey's *post hoc* test and between multiple comparisons at multiple time-points, repeated measures anova was used. A *P* < 0.05 was considered statistically significant.

##### Serum PDGF level

At 60 DAI, treatment with BPG significantly increased serum level of PDGF-AA and AB compared to the control, CI and CI-PDS-treated groups (*P* = 0.001 for all). At 120 DAI, the serum PDGF level significantly decreased compared to 60 DAI (*P* = 0.001 for all), but the BPG-treated animals had still significantly higher serum PDGF-AA and -AB level when compared to the controls (*P* = 0.001 for all; Table2).

#### Post-euthanasia assessments

##### Gross pathology

Gross pathological evaluations were performed immediately after euthanasia. Treatment with BPG significantly enhanced the scoring values for the development of peritendinous adhesion (*P* =0.001, *P* = 0.001, *P* = 0.042), hyperaemia (*P* = 0.001, *P* = 0.001, *P* = 0.002), general appearance of the neotenon (*P* = 0.001, *P* =0.001, *P* = 0.026), muscle atrophy (*P* = 0.001, *P* = 0.001, *P* = 0.001) and muscle fibrosis (*P* = 0.001, *P* = 0.001, *P* = 0.003) compared to the control, CI and CI-PDS-treated groups respectively (Table1).

##### Histopathology

At 60 DAI, treatment with BPG significantly reduced total cellularity, total fibroblast, immature fibroblast and lymphocyte but also it significantly increased number of mature fibroblast and fibrocyte, macrophages, small-, medium- and large-sized blood vessels, and collagen density compared to control (defect) tendons (*P* = 0.001 for all). At this stage, the BPG-treated tendons had significantly higher cellularity, total fibroblast, mature fibroblast, fibrocyte, macrophages, total vessels, medium- and large-sized blood vessels, diameter of medium and large-sized blood vessels and collagen density and also had significantly lower immature fibroblast, small-sized blood vessels, and cell transverse diameter compared to those tendon treated with either the CI or CI-PDS (*P* = 0.001 for all). At 120 DAI, cellularity, total fibroblast, number of immature fibroblast, inflammatory cells, number of small-sized blood vessels, and cell density significantly decreased in all the groups when compared to 60 DAI (*P* < 0.05). At this stage, those tendons treated with BPG had significantly less total cellularity, immature fibroblast, mature fibroblast, lymphocyte, macrophage, small- and medium-sized blood vessels, and cell density and also they had significantly higher fibrocyte, transverse diameter of the medium- and large-sized blood vessels, and collagen density compared to the control (defect), CI and CI-PDS-treated tendons (*P* = 0.001 for all; Table3). Treatment with BPG significantly enhanced the scored values for tissue alignment (*P* = 0.001 for all), perivascular oedema (*P* = 0.001 for all), tissue maturity (*P* = 0.001, *P* = 0.001, *P* = 0.017), crimp pattern (*P* = 0.001, *P* = 0.001, *P* = 0.002), and vascularity (*P* = 0.001 for all) compared to the control, CI and CI-PDS-treated groups respectively (Table1, Fig.5).

**Table 3:** Histopathological and histomorphometric analyses of the injured tendons after 60 and 120 days of tendon injury and surgical operation

	60 days after tendon injury				120 days after tendon injury			
	Control	CI	CI-PDS	CI-PDS-PG	Control	CI	CI-PDS	CI-PDS-PG
Number of total cell	523.41 ± 19.14	373.44 ± 8.01	401.2 ± 9.59	428.64 ± 7.44	443.11 ± 16.37	289.17 ± 8.36	273.71 ± 7.59	236.62 ± 11.3
Number of total fibroblast	334.24 ± 10.9	257.06 ± 5.31	241.17 ± 9.1	280.21 ± 5.33	322.14 ± 15.19	222.48 ± 9.68	191.54 ± 7.91	217.41 ± 8.85
Number of immature fibroblast	221.82 ± 10.83	96.41 ± 5.63	85.70 ± 5.07	49.10 ± 5.96	167.48 ± 12.11	68.73 ± 6.37	41.05 ± 5.36	28.39 ± 5.24
Number of mature fibrocyte	111.44 ± 6.43	144.68 ± 8.99	131.82 ± 6.11	195.60 ± 12.33	121.44 ± 11.34	122.91 ± 3.84	109.22 ± 6.58	96.46 ± 6.07
Number of fibrocyte	3.72 ± 1.64	17.15 ± 2.64	24.94 ± 3.28	44.56 ± 3.05	22.01 ± 2.53	33.31 ± 2.69	46.31 ± 4.65	84.8 ± 5.32
Number of neutrophil	7.29 ± 1.73	14.18 ± 2.04	23.47 ± 3.66	29.31 ± 4.75	1.09 ± 0.69	0.97 ± 0.63	4.08 ± 2.58	3.33 ± 1
Number of lymphocyte	87.31 ± 7.4	51.16 ± 4.3	66.44 ± 5.31	59.58 ± 6.59	63.51 ± 5.6	39.63 ± 5.2	46.34 ± 3.44	15.81 ± 1.93
Number of plasma cell	4.19 ± 1.54	2.67 ± 1.3	12.54 ± 2.24	10.19 ± 1.37	2.56 ± 1.12	1.15 ± 0.87	1.84 ± 0.92	1.34 ± 0.62
Number of macrophage	59.32 ± 8.19	43.23 ± 3.89	53.67 ± 3.19	66.3 ± 2.54	33.22 ± 5.67	22.44 ± 2.64	24.21 ± 2.95	7.55 ± 0.95
Number of vessels	12.35 ± 2.96	33.44 ± 3.93	25.19 ± 3.65	51.85 ± 4.36	32.87 ± 6.26	16.9 ± 3.28	11.06 ± 1.27	7.24 ± 0.81
Number of small vessels	7.84 ± 0.63	26.7 ± 2.02	19.88 ± 1.29	15.48 ± 1.45	17.29 ± 2.24	10.16 ± 0.98	6.32 ± 1	1.34 ± 0.6
Number of medium vessels	3.07 ± 0.88	3.67 ± 0.7	5.17 ± 1.01	16.22 ± 1.45	6.83 ± 0.75	4.72 ± 0.65	3.38 ± 0.38	2.33 ± 0.35
Number of large vessels	0.62 ± 0.56	3.09 ± 0.98	5.05 ± 1.39	12.52 ± 1.93	4.74 ± 1.65	3.1 ± 0.93	1.94 ± 0.66	4.3 ± 1.72
Diameter of immature fibroblast (μm)	7.93 ± 1.23	6.72 ± 0.87	6.29 ± 0.43	5.06 ± 0.61	7.82 ± 1.13	5.49 ± 0.33	5.55 ± 0.36	4.88 ± 0.51
Diameter of mature fibroblast (μm)	3.7 ± 0.15	3.35 ± 0.18	3.57 ± 0.14	2.76 ± 0.37	3.31 ± 0.39	2.79 ± 0.36	2.55 ± 0.2	2.49 ± 0.19
Diameter of fibrocyte (μm)	1.69 ± 0.04	1.32 ± 0.04	1.3 ± 0.03	1.2 ± 0.02	1.52 ± 0.08	1.24 ± 0.05	1.18 ± 0.02	0.97 ± 0.03
Diameter of small vessels (μm)	11.9 ± 0.86	15.64 ± 1.03	15.36 ± 0.82	24.43 ± 1.98	15.24 ± 0.97	21.6 ± 1.93	25.13 ± 1.09	28.32 ± 1.19
Diameter of medium vessels (μm)	34.41 ± 1.58	42.9 ± 2.65	45.29 ± 2.24	54.18 ± 2.13	42.57 ± 1.85	50.48 ± 1.65	51.03 ± 2.08	56.86 ± 2.18
Diameter of large vessels (μm)	69.19 ± 4.08	82.47 ± 3.68	92.6 ± 3.61	119.01 ± 5.25	81.62 ± 4.31	101.04 ± 3.57	122.31 ± 5.51	192.9 ± 8.05
Percentage of collagen density	8.21 ± 1.86	35.19 ± 3.3	45.08 ± 1.98	60.52 ± 3.32	23.52 ± 4.03	62.38 ± 3.02	70.65 ± 3.76	86.99 ± 4.69
Percentage of cell density	24.77 ± 3.4	15.81 ± 0.98	21.49 ± 2.32	21.32 ± 2.42	19.86 ± 1.65	10.62 ± 1.36	15.19 ± 1.47	9.23 ± 0.84
Percentage of background density	63.16 ± 2.27	49.63 ± 2.98	35.4 ± 2.16	15.98 ± 1.24	48.46 ± 2.9	32.9 ± 2.84	21.65 ± 3.02	11.6 ± 1.27

All the quantitative values were expressed as mean ± SD. The significant differences in the measured values between multiple groups at one time-point (*e.g*. 60 DAI) were tested using one-way anova with subsequent Tukey's *post hoc* test and between multiple comparisons at multiple time-points, repeated measures anova was used. A *P* < 0.05 was considered statistically significant. CI: collagen implant; PDS: polydioxanone; PG: platelet gel. For the histopathological analyses, 20 tendon samples × 3 tissue sections × 5 histological field has been used in each group so that totally 300 histopathological micrographs were evaluated.

##### Scanning electron microscopy

At 60 and 120 DAI, treatment with BPG significantly increased transverse diameter and density of the collagen fibrils, fibres and fibre bundles and also it sooner differentiated the collagen fibrils to fibres and fibres to fibre bundles compared to the controls (*P* = 0.001 for all; Table4). In addition, treatment with BPG significantly improved the scored values for tissue alignment (*P* = 0.001, *P* = 0.001, *P* = 0.033), maturity of the collagen fibrils (*P* = 0.001, *P* = 0.001, *P* = 0.048) and crimp pattern (*P* = 0.001, *P* = 0.001, *P* = 0.002) compared to the control, CI and CI-PDS-treated groups respectively (Table1, Fig.5).

**Table 4:** Scanning electron microscopic features of the injured tendons after 60 and 120 days of tendon injury and surgical operation

	60 days after tendon injury				120 days after tendon injury			
	Control (*n* = 20)	CI (*n* = 20)	CI-PDS (*n* = 20)	CI-PDS-PG (*n* = 20)	Control (*n* = 20)	CI (*n* = 20)	CI-PDS (*n* = 20)	CI-PDS-PG (*n* = 20)
Transverse diameter of collagen fibrils (nm)	29.52 ± 3.27	46.21 ± 4.15	57.76 ± 3.88	64.45 ± 4.92	39.2 ± 5.02	61.92 ± 3.92	68.41 ± 6.57	82.62 ± 6.59
Transverse diameter of collagen fibres (nm)	0	456.84 ± 14.46	512.01 ± 17.95	614.35 ± 27.32	525.14 ± 10.75	546.45 ± 15.96	542.01 ± 25.16	644.2 ± 17.47
Transverse diameter of collagen fibre bundles (nm)	0	0	0	3881.93 ± 398.15	0	2864.75 ± 330.34	4332.09 ± 610.56	6161.07 ± 571.39
Number of cells	257.33 ± 11.63	187.54 ± 7.55	172.53 ± 9.04	148.17 ± 7.5	195.71 ± 9.75	75.72 ± 5.57	69.06 ± 6.51	57.22 ± 4.96
Percentage of fibrils density	33.24 ± 4.43	58.88 ± 3.81	65.1 ± 2.35	71.85 ± 3.57	47.03 ± 4.88	65.76 ± 3.9	72.96 ± 3.6	76.71 ± 5.14
Percentage of Cell density	22 ± 3.3	18.86 ± 1.65	16.45 ± 1.44	12.52 ± 1.11	17.06 ± 1.25	9.97 ± 1.64	7.9 ± 1.13	7.53 ± 1.25

All the quantitative values were expressed as mean ± SD. The significant differences in the measured values between multiple groups at one time-point (*e.g*. 60 DAI) were tested using one-way anova with subsequent Tukey's *post hoc* test and between multiple comparisons at multiple time-points, repeated measures anova was used. A *P* < 0.05 was considered statistically significant.

##### Host–graft interaction mechanism

**Figure 6:**
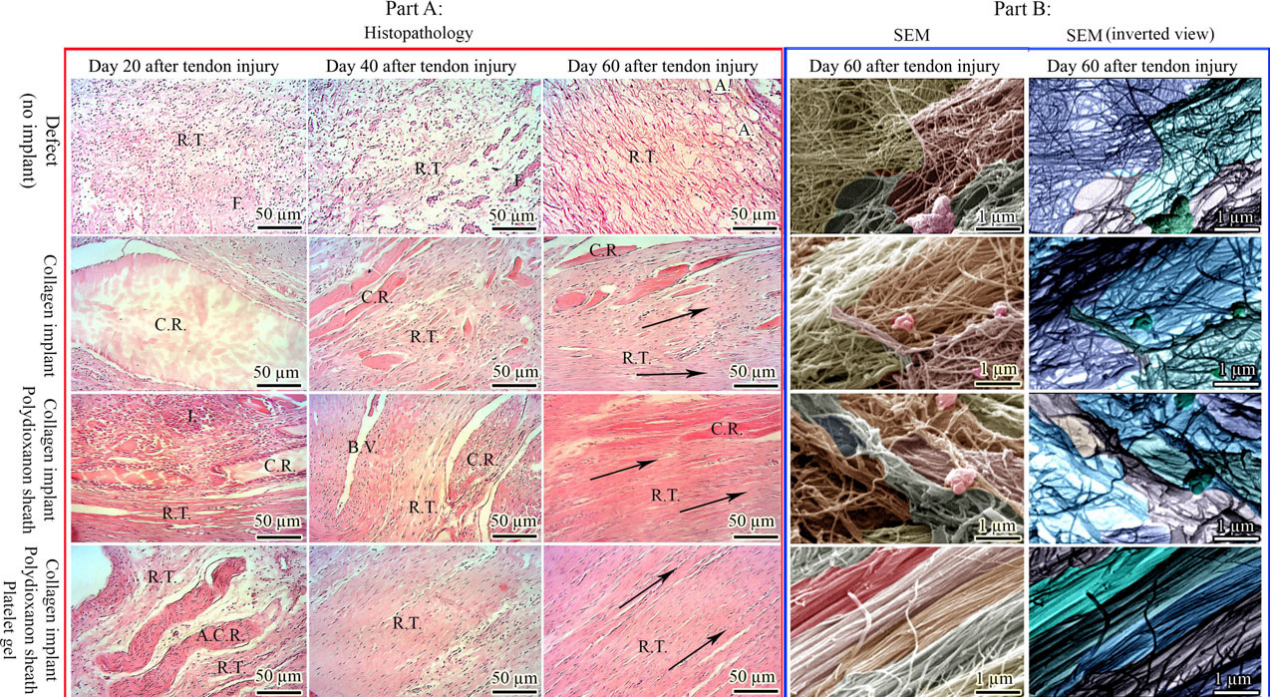
Host–graft interaction mechanism. (A) Histological view: Arrows show the direction of the collagenous tissue regenerated in the defect area. Note that no characteristic healing response has occurred in the defect group. The collagen remnants are present even after 60 days of tendon injury in the collagen and collagen-PDS-treated groups. However, some of them were accepted as a part or gradually absorbed by the host inflammatory cells. Bovine platelet gel considerably improved the biocompatibility of the implant so that most collagen remnants were accepted as a part of the neotenon and incorporated with the healed tendon so that after 60 days no implant remnant could be seen. (B) SEM view: the collagen fibrils have been shown. Bovine platelets ultrastructurally affected tendon healing and produced a highly remodelled tissue so that its collagen fibrils diameter, arrangement and density is comparable to normal tissue. Other groups have inferior morphological characteristics than those tendons treated with bovine platelets. R.T: regenerated tissue; A.C.R: accepted collagen remnant; C.R: collagen remnant; I: inflammation; F: fibrin, A: Adipose tissue.

Based on different time-point evaluations performed after tendon injury, actually no characteristic inflammation, fibroplastic response and healing occurred in the control (defect) tendons. Only a loose areolar connective tissue similar to the surrounding fascia filled the defect area. After implantation of various prosthetic implants in tendon defect, the implants increased inflammation and transverse diameter of the injured area at short-term and this accelerated a strong fibroplastic response so that a new granulation tissue covered the implant at periphery and gradually invaded inside the implant. At microstructure, a demarcation line between inflammatory cells and internal parts of the CI and CI-PDS implants was seen. At later stages, the inflammatory cells degraded the implants but several pieces of collagen remnants were still present in the injured area even after 60 days. Some few remnants were accepted as a part, some others gradually degraded and the rest of them were surrounded by the fibrous connective tissue and were present in the new tendon. Grossly, the implants were degraded and the new tendon replaced the implants after 40 days. In contrast to the CI and CI-PDS-treated tendons, those tendons treated with BPG had no demarcation line at inflammatory stage and the inflammatory cells well distributed all over the implant. The BPG-treated group showed higher fibroplastic response and the neotenon replaced the implant after 20 days. The collagen remnants were not present after 60 DAI because they absorbed or accepted as a part of the neotenon sooner than the other groups (Fig.6).

##### Dry matter and hydroxyproline contents

**Figure 7:**
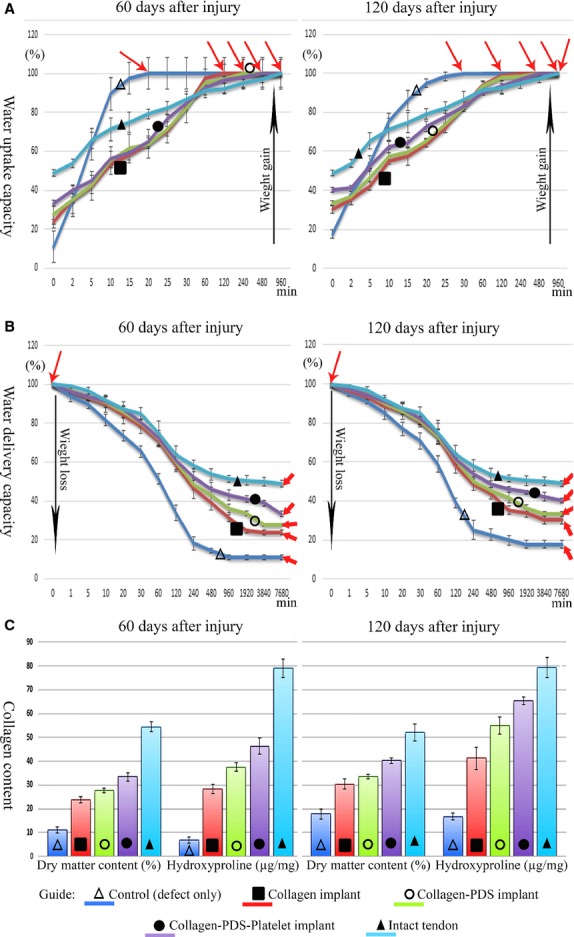
Water uptake, water delivery, hydroxyproline and dry matter contents of the injured and normal tendons after 60 and 120 days post injury. Note that, compared to the controls, the bovine platelet gel + artificial tendon has a closer pattern of water uptake (A) and delivery (B) capacity to normal tendon and has higher dry matter content and hydroxyproline (C) compared to controls (*P* < 0.05 for all). In another meaning, the treated tendons with bovine platelet gel, similar to normal tendons, absorb hydration slowly (A) and deliver (B) it slowly and have significantly higher collagen content (C) than controls (*P* < 0.05). Thin arrows show wet weight and thick arrows show dry weight.

Dry matter and hydroxyproline contents are indices of total collagen content of a tissue. Treatment with BPG significantly increased dry matter and hydroxyproline contents of the injured tendons compared to the control, CI and CI-PDS-treated tendons at 60 and 120 DAI (*P* = 0.001 for all). Compared to 60 DAI, at 120 DAI, these factors significantly increased in all the groups (*P* = 0.001 for all). Although bovine platelets improved dry matter and hydroxyproline contents of the injured tendons, however at 120 DAI, these factors were still significantly lower than the normal healthy tendons (*P* = 0.001 for all; Fig.7).

##### Water uptake and delivery capacities

Those tendons treated with various prosthetic implants had more similar pattern of water uptake and delivery compared to control tendons (defect). Among the treated tendons, those treated with BPGs had a closer behaviour of water uptake and delivery to normal tendons than the CI and CI-PDS-treated tendons. The fully dehydrated platelet-treated tendons absorbed the hydration and reached their maximum wet weight after 960 min. which was similar to normal tendons, but the CI and CI-PDS-treated tendons reached their wet weight after 120 min. and the control tendons reached their maximum wet weight after only 30 min. In addition, the fully hydrated platelet-treated tendons, fully delivered their total hydration and reached their final dry weight after 7680 min. which was similar to normal tendons, while the CI and CI-PDS-treated tendons and the control tendons reached their dry weight after 3840, 3840 and 1920 min. respectively. Those tendons treated with BPGs and also the normal tendons had significantly less index of water uptake and delivery compared to the CI and CI-PDS-treated and control tendons (*P* = 0.001 for all; Fig.7).

## Discussion

The results of the present study showed that the BPGs when embedded within CI-PDS (artificial tendon) significantly enhances cytocompatibility of the implant at *in vitro* level but also improves scaffold biocompatibility and biodegradability *in vivo*. BPGs increased inflammation at short-term but produced a strong fibroplastic response at midterm; thus, enhanced neotenon formation and tendon remodelling and maturation at later stages.

Inflammation is an important part of tendon healing 7,17,36. Without a proper inflammatory response, no proper healing and regeneration could be expected as we observed in our control groups (defect, no implant). Insertion of various prosthetic implants in the defect area, increased inflammation thus triggered fibroplasia. BPG significantly increased inflammation than controls as demonstrated by measuring the transverse diameter of the injured area, surface temperature, clinical evaluations and gross and histopathological analyses; however, the inflammation did not exaggerated and also its duration was modulated by the BPG. In fact, the BPG accelerated the rate of inflammatory response so that it diminished the inflammation sooner than the CI and CI-PDS from days 14 to 21 after injury. Galliera *et al*. 25 showed that platelets play an active role in the immunological and inflammatory aspect of tissue healing. Indeed, they can be directly involved in the inflammatory response by the production and release of several inflammatory mediators. Platelets are not only a source of several chemokine involved in the inflammatory response and tissue healing but they also express chemokine receptors, thus being able to regulate the inflammatory response associated to the healing process. During inflammation, neutrophils, macrophages and lymphocytes infiltrate the injured area 7. Histological evaluations suggest that platelets caused better inflammatory cell distribution all over the implant. Unlike the platelet-treated group, the inflammatory cells failed to expose with the inner parts of the implant in the CI and CI-PDS-treated groups; thus, a demarcation line was formed between the CI and CI-PDS implants and the host soft tissues. In the CI and CI-PDS groups, a fibrous connective tissue surrounded the implants and gradually incorporated within them so that the inflammatory cells gradually invaded the more internal parts of the implants, time dependently. Therefore, some islands of collagen remnants were diagnostic even after 60 days of injury. Unlike them, in the BPG-treated group, the inflammatory cells exposed with the internal parts of the implants and the fibrous connective tissue sooner regenerated in those parts when compared to the other groups thus we concluded that the biocompatibility of the CI was enhanced by the platelets. During inflammation, macrophages are regulated by lymphocytes 8. T helper type I lymphocytes regulate macrophages type I which are inflammatory in nature and cause graft rejection 7,31. T helper type II lymphocytes regulate macrophages type II which have remodelling effect and cause graft acceptance 31. In comparison between those that were treated with platelet-implant and those treated with implant only, the results suggest that the BPGs trigger type II pathway because bovine platelets increased graft acceptance rate as demonstrated by infiltration of the healing fibroblasts in the collagen islands after the inflammation terminated. Bovine platelet gel also increased the biodegradability of the implants more than that observed in the other groups because no remnants of the CI or the PDS sheath were seen after 60 days of tendon injury.

Bovine platelet gels also produced a stronger fibroplastic response compared to the controls. One possible mechanism may be because of the role of bovine platelets in increasing the inflammation and in triggering the type II inflammatory pathway. It has been shown that remodelling macrophages (type II) facilitate the transition between inflammation to fibroplasia 8,31. We showed without inflammation, no effective fibrous connective tissue invade the wound site as seen in the control group (defect only). BPGs increased fibroblasts proliferation and maturation, enhanced their distribution and improved their matrix production both *in vitro* and *in vivo*. This is the tenoinductive properties of bovine platelets 31. Possibly, bovine platelets enhanced fibroplasia because of their effectiveness on angiogenesis and angiomaturation 4. Bosch *et al*. 3 studied the role of PRP on neovascularization of experimentally induced tendon injuries in horse showing single injection of PRP is able to significantly increase neovascularization of the healing tendon. Also, Vogrin *et al*. 27 in a prospective, randomized, double-blind, clinical trial study showed that locally applied platelet gel enhances early revascularization of the graft in the osteoligamentous interface zone after ACL reconstruction.

Bovine platelets also enhanced tenoconductivity of the implants because as the graft gradually absorbed, the neotenon gradually formed according to the anatomical line of the tendon. This decreased the development of peritendinous adhesion and muscle fibrosis, thus concentrated the healing response only at the wound site 8. These beneficial results may also be because of the role of platelet-derived growth factors 14,18. Zhang and Wang 37 showed PRP-clot releasate because of its growth factors promotes differentiation of tendon stem cells into active tenocytes exhibiting high proliferation rates and collagen production capability. Also, Hoppe *et al*. 18 showed, tenocytes of chronic rotator cuff tendon tears can be stimulated by platelet-released growth factors so that the growth factors increased cellular proliferation, migration, differentiation and matrix production *in vitro* which is similar to our *in vitro* results. In addition, Anitua *et al*. 32 showed that releasates from both platelet-rich and platelet-poor clots stimulated tendon cell proliferation, in contrast to un-clotted PPP. Cultured tendon cells synthesized VEGF and HGF in the presence of PPP-clots and PRP-clot releasates, thus the synthesized amount was significantly higher with supernatants from platelet-rich clots than supernatants from a platelet-poor clot. We measured the PDGF level of the animals and interestingly found that bovine platelets increased this growth factor in the treated animals more than the controls suggesting the better quality of tendon healing has been achieved. It should be highlighted that serum PDGF level is a valuable index of tendon healing 8,13. Van den Dolder *et al*. 38 showed that PRP has considerable amounts of all the three isoforms of PDGF-AA, -BB and -AB. Lepistö *et al*. 39 demonstrated that PDGF-AA and PDGF-BB down-regulated both the steady-state levels of pro-alpha 1 (I and III) collagen chain mRNAs and the production of collagen, in a dose-dependent manner. Interestingly, PDGF-AB up-regulated the expression of type I and III procollagen mRNAs by cultured wound fibroblasts. Pierce *et al*. 40 detected little PDGF isoform expression in normal skin and in non-healing dermal ulcers. In contrast, in surgically produced acute wounds and chronic ulcers treated with rPDGF-BB, markedly up-regulated levels of PDGF-AA were found. In both types of wounds, increased PDGF-AA was detected primarily in capillaries and fibroblasts. While PDGF-BB were present in a minority of healing wounds, they were usually present at lower levels than PDGF-AA. It seems PDGF-AA may be a superior indicator of wound healing, while PDGF-BB may have greater therapeutic value, and the AB type may be both therapeutic and diagnostic.

At macro, micro and ultrastructure, the BPGs improved tendon texture, collagen fibre density and alignment, collagen fibrils number, diameter, density and continuity respectively. Our results suggest that these morphological features are in strong positive correlation with hydroxyproline and dry matter contents and all of these, are indices of collagen content 8,13,41. Sadoghi *et al*. 22 in a systematic review on effectiveness of autologous platelets (*e.g*. PRP) on healing and repair of tendon ruptures and tendinopathies in human and animal models, concluded that platelets are effective in tendon ruptures because they have a role on tissue maturation and collagen production.

Bovine platelets by the above mechanisms accelerated remodelling phase of tendon healing because the BPG-treated group had more mature fibroblast and fibrocyte, higher degree of collagen fibril and fibre alignment, collagen fibril to fibre and collagen fibre to fibre bundle differentiation and finally had a more organized structure than controls. Determination of water uptake and delivery are indices of tissue organization 8,31. It has been shown that, organized tissues such as intact tendon absorb and deliver the hydration slowly 8. Those tendons treated with BPG had a closer behaviour of water uptake and delivery to intact tendons when compared to the controls. All these mechanisms caused a better and superior clinical results observed in the platelet-treated group. In fact, the BPG-treated animals had better ultrasonographical features (*e.g*. echogenicity and homogenicity) and had better weight bearing and physical activity than controls. Less muscle atrophy of the treated animals than the controls could be because of the better clinical outcome and the ability of the neotenon to do its normal physiological function which has a great value in the clinical setting 7. Tendons connect muscle to bone and allow transmission of forces generated by muscle to bone, resulting in joint movement. Following tendon injury, the continuity of muscle–tendon–bone unit is impaired so that joint function significantly decreases resulting in inappropriate work of the associated muscle. Therefore, muscle atrophy is developed 31. By surgical reconstruction of a raptured tendon, this continuity is established but the function is not immediately improved 7,41. After injury, the functionality of the muscle is dependent to the quality of tendon healing 5,41. If tendon healing had poor quality, then muscle fibrosis and peritendinous adhesions develop because of improper proliferation of the healing fibroblasts in all directions 8. Normal healthy paratenon in extra synovial tendons and tendon sheath in intra-synovial tendons have great role in guiding the fibroblasts to proliferate inside the injured area and in inhibiting their proliferation to the surrounding tissues 7,42. Unfortunately, after tendon injury, the paratenon continuity is altered so that its proliferating fibroblasts extend to the associated muscle and surrounding tissues and produce a large amount of collagenous matrix which is organized by time resulting in adhesion of the tendon to the surrounding tissues and development of muscle fibrosis 7,8. In such condition, the healing quality is impaired because a considerable number of fibroblasts produced collagen matrix outside the injured area. These adhesions together with muscle fibrosis inhibit tendon gliding movement in its normal anatomical space resulting in development of muscle atrophy 7,8. In the present experiment, we removed 20 mm of the Achilles apparatus together with the covering paratenon so that after the healing started, no paratenon existed to guide the healing fibroblasts to proliferate inside the tendon defect area thus the healing fibroblasts proliferated in all over the directions, produced collagenous matrix between tendon edges and the surrounding tissues. In addition, some of them were migrated to the adjacent gastrocnemius–soleus muscle, and produced collagen matrix between the muscle fibres resulting in development of muscle fibrosis. Because no tendinous tissue formed in the defect area of the control group, the gastrocnemius–soleus muscle–Achilles apparatus–calcaneal joint (GSM-AA-CJ) function did not stablish and therefore the muscle atrophy was developed. This finding was correlated with the improper weight bearing and physical activity of the control (defect) animals. Unlike the controls, in the treated groups with CI, CI-PDS and CI-PDS-BPG, the bioimplants attracted the healing fibroblasts originated from both the paratenon and tendon edges to their internal architecture, guided them according to the anatomical line of the GSM-AA-CJ system, and enhanced their proliferation and collagen synthesis. Compared with the CI and CI-PDS bioimplants, the CI-PDS-BPG bioimplant amplified these beneficial effects, thus concentrated the healing fibroblasts in the injured defect area resulting in formation of a new tendinous tissue while inhibiting development of peritendinous adhesions and muscle fibrosis. Therefore, treatment with BPG, established the function of the GSM-AA-CJ system, controlling and inhibiting development of gastrocnemius–soleus muscle atrophy. These findings are in accordance to the superior weight bearing and physical activity of the treated animals when compared to the controls. These beneficial physical performance observed in the treated animals had further role during tendon healing because when the working of the GSM-AA-CJ system increased; the tendon morphological and biophysical properties also improved. One of the beneficial role of working GSM-AA-CJ system is that by increasing in the physical activity on the injured limb and accordingly, increasing in the transmission of force through the healing tendon, the healing newly regenerated collagen fibrils are aligned in one direction thus differentiated and packed as collagen fibres which have a great role in further increasing the functional performance of the healing tendon 7,42.

The results suggest that BPG is a safe agent because it restored the haematological profile of the animals faster compared to controls. There are many studies reported, suggested and recommend PRP as an effective therapeutic approach for musculoskeletal injuries 4,16. Recent trends suggested controversy in the use of autogenous form of platelets in tendon or other musculoskeletal tissue injuries. For example, Visser *et al*. 20 implanted autologous platelet-rich fibrin (PRF) membrane in patellar tendon defect model in eight dogs and showed a PRF membrane does not enhance the rate and quality of tendon healing in patellar tendon defects. However, it did increase the amount of repair tissue within and surrounding the defect. In another study, Schepull *et al*. 28 in a randomized controlled trial study (Level of evidence, 2), showed that PRP is not useful for treatment of Achilles tendon ruptures. The mechanical variables showed a large degree of variation between patients that could not be explained by measuring error. No significant group differences in elastic modulus could be shown. There was no significant difference in heel raise index. The Achilles Tendon Total Rupture Score was lower in the PRP group, suggesting a detrimental effect.

Animal studies have used allogenous form of PRP, showing superior results compared to the clinical investigations. Aspenberg and Virchenko 19, in a study in rat Achilles tendon defect model showed, after intra-haematoma injection of allogenous concentrated platelets, tendon callus strength and stiffness increased by about 30% after 1 week, which persisted for as long as 3 weeks after the injection. Histology showed treatment improved tendon callus maturation. Also, Beck *et al*. 43 showed that allogenous PRP is effective in the healing and regeneration of tendon-from-bone supraspinatus tear model in rats because it increased fibroblastic response and vascular proliferation during the first 21 days of tendon injury, but failed to improve mechanical strength of the healing tendons more than that observed for the controls. Matsunaga *et al*. 12 using the same methodology as ours, but using allogenous compact platelet-rich fibrin scaffold (CPFS), implanted the CPFS in the injured area of the partial patellar tendon defect and ligament reconstruction models in rabbits showing after 12 weeks, the ultimate failure load and stiffness are higher for the right patellar tendon than for the left patellar tendon (control, no implant). In the former model, it was found that CPFS promoted ligament repair tissue in contrast with that on the untreated side in the latter model. Finally, Lyras *et al*. 36 in an experimental study in rabbit patellar tendon defect model showed application of allogenous platelet gel in the defect area is able to significantly increase the mechanical properties of the healing tissue during the first 28 days of tendon injury and it appears that allogenous PRP has a strong effect in the early-phase of tendon healing. This effect is probably because of the growth factors that are released from the platelets during activation.

To date, there are few studies which used xenogenous-based platelets for tissue healing. In a series of relevant studies, it has been shown that xenogenous-based human platelets have promising curative effects on healing and regeneration of rabbit radial bone defect model 15,29,30. Xenotransplantation is not a popular option in the clinical practice. Historically, most of the xenografts tissues (*e.g*. kidney, liver, heart, *etc*) were transplanted with the aiming to establish organ function 44–49. Clinical experiences strongly showed that after xenogenous transplantation of such solid organs, the transplanted tissue would be rejected acutely or chronically even if the immune system aggressively suppressed. In such cases, severe fibrosis, necrosis or massive degradation would lead to graft failure 44–46. In the recent years, by developing new and novel technologies in tissue engineering and regenerative medicine, xenogenous tissues have been processed in a manner to be used as tissue scaffolds and this is differ with organ transplantation 47–49. Extensive experiences exist in this issue, showing xenogenous transplantation of processed collagenous materials could be well tolerated by the host 11,47–49. For collagenous-based materials for example, acellularization technologies have been used to remove the xenogeneic cells with the aiming to reduce inflammatory reaction in response to xenogenous transplantation of a processed graft 6–11. In the present study, we used BPG as a xenogeneic source for the platelets to enhance tendon healing quality. In this specific case, we removed bovine red blood cells and white blood cells as the major antigenic sources of the BPG and therefore reduced potential immunological reactions in the rabbit body. In addition, BPG was not a slow degradable product and could not trigger the inflammatory reaction for a long period of time as we showed in the present study that the inflammation declined after 2 to 3 weeks of implantation of the CI-PDS-BPG suggesting that the BPG although increase the inflammation for a short period of time but such inflammation was not exaggerated and also it was not prolonged. Such duration of the inflammatory response is a consequence of an effective tendon healing and is normally happened after surgery 7. Therefore, for the first time, we showed it is possible to use xenogenous-based platelets for tendon healing.

Further studies are needed to answer whether the effectiveness of allogenous and xenogenous platelets in wound healing has correlation with platelet growth factors or is because of the role of platelets on inflammation. The results of this comprehensive study showed that both mechanisms may be involved. In fact, the BPGs both increased the inflammation at short-term and also increased the serum growth factor levels at different time-points which we think it may be because of the released platelet-derived growth factors of the BPGs at the wound site.

Simple surgical techniques are often used to reconstruct small tendon defects 1,2,5,7. End-to-end repair is ideal if the gap between tendon ends allow direct apposition after resection of the interposed scar tissue allowing for maximum isokinetic strength of Achilles because re-establishment of the pre-injury tendon length can only be achieved 5. Primary repair is still an uncommon treatment strategy for most chronic ruptures and large defects because of the potential for shortening and contracture of the GSM-AT unit 1,2. Excision of scar tissue from neglected rupture often results in a sizable gap requiring other modalities to bridge the defect. If the gap exceeds 1–2 cm (in clinical patients) and primary repair is still deemed feasible, proximal lengthening of the gastroc-soleus complex may be used to achieve mobilization of the proximal tendon end to facilitate primary repair 1,2,5. In large tendon defects, tendon transfer, free transfer, pedicled flap and other methods have been utilized to reconstruct the defect area 1,2. V-Y advancement flap and flexor halluces longus tendon transfer have been found to be reliable and achieve good clinical outcome for medium-sized defects in clinically ill patients (2–8 cm) 50. These techniques have their own limitations 5. Increasing the surgical time, injury to the normal tissues, pain, need for the extensive exposure, risk of reinjury and dehiscence are some examples for each of these techniques 51. In addition, most of these methods are modified methods used by different surgeons based on the defect size, condition of the patient and experience of the surgeon 52.

In comparison between available classic grafts including auto-, allo and xenografts, using autografts is more reliable and effective approach for large tendon defect reconstruction and allografts are alternative option to autografts so that the fresh, and processed models of allografts are available for tendon reconstruction 7. We introduced and mentioned the limitations of these options at introduction section. In addition to their significant limitations, although it is possible to enhance the healing capability of the classic grafts by combining them with PRP therapy or other medications, because it is not possible to reorganize the internal architecture of the grafts, they would not be an optimum scaffold for tendon regeneration and could not produce an align and uniform neotenon 7,53. It is a fact that all of the grafts, even vascular grafts are partially degraded after transplantation in the recipient site, therefore, the biomechanical properties of the newly regenerated tendon in the degraded part of the graft has significant differences with the accepted parts of the graft and compared to our strategy, the amount of graft in the new tendon is higher in the classic graft option 7,53.

Altogether, despite of modern technologies used to process the allografts, many surgeons still prefer to use autografts for large tendon reconstruction 53. Maffulli *et al*. 54 showed that minimally invasive ipsilateral semitendinosus reconstruction of large chronic Achilles tendon tears may be advantageous for the patient in terms of plantar flexion recovery, anthropometric measures, fast return to daily and sport activity, is safe, with low donor site co-morbidities, low risks of wound complications and neurovascular injuries. Also Takeuchi *et al*. 55 used semitendinosus tendon for large Achilles tendon reconstruction with favourable short outcome. Although classic grafts are the available option, the overall risk of complications is high and in particular the risk for wound healing problems, which are triggered by an increased tension in the skin when inserting a bulky graft to cover the rupture 51.

There are a number of commercially available extra cellular matrices (ECM) for tendon reconstruction 7. These are including TissueMend (source: foetal bovine), SergiMend (source: foetal or neonatal bovine), Primatrix (source: foetal bovine), Permacol, Zimmer collagen patch, Collamend, Conexa and Strattice (source: adult porcine), Alloderm, GraftJacket, Flex HD, Allopatch, Allomax and Neoform (source: adult human), which are all produced from dermis layer of the skin, Restore, Surgisis Biodesign, Oasis and CuffPatch (source: adult porcine) which are produced from small intestine submucosa, OrthAdapt and Unit which are produced from pericardium of adult equine and finally, Veritas and Peri-Strips which are produced from pericardium of adult bovine 56. Although all of them are collagen-based matrices, they have significant differences with the CI-PDS-BPG construct which we introduced in the present investigation. First of all, these matrices are produced using acellularization technology so that only the cells or other micro-organisms could be removed from the ECM by this technology 7,56. Perhaps, other antigenic molecules related to ECM may trigger inflammation 10,47–49. Second, the orientation of the collagen fibres could not be changed so that it is not possible to reorganize the internal architecture of the scaffold 7,10. Third, Most of these scaffolds are two dimensional in nature; it means they are patch like ECMs and could not be considered as alternative to three-dimensional tissue grafts 7,56. Forth, Most of them are chemically cross-linked with toxic agents and the cross-linking degree is different between different products 56. In addition, some of them are normally sterilized with ethylene oxide gas which is a toxic technology and the gas may be present at cellular level even after several days of air exposure 56. Finally, despite of extensive researches provided preliminary data regarding commercial ECMs, few investigations showing the exact mechanisms of their degradation, tissue incorporation, and healing capability are present in the literature 7,10,47–49. Because of the variation in the cross-linking methods, haphazardly orientation of the collagen fibrils, and two-dimensional architecture of these ESM-based implants, they are not suitable options for reconstruction of large tendon defects 56. Most of the commercially available ECM-based implants routinely receive high degrees of cross-linking which decreases their biodegradability and consequently increases their presence time in the host body. This extends the inflammatory period to several weeks after surgical implantation 56. In such condition, the graft may be present partially even after months, thus tendon rupture could be occurred just from those parts that the bioimplant is present in the injured healing tissue 47–49. This greatly increases the risk of dehiscence so that revision surgery may be required 7,53. In addition, based on their two-dimensional structures, these bioimplants are most often used as patch for reconstruction of small tendon defects or may be used as a barrier for development of peritendinous adhesions 7. Such scaffolds are often used for augmentation of rotator cuff tendon ruptures or as a tendon sheath for hand flexor tendons and are not suitable for large tissue defects similar to the model we presented in this study (*e.g*. large Achilles tendon defect model). Giannotti *et al*. 57 used porcine dermal collagen patch (PDCP) in 25 patients who underwent surgery to treat massive symptomatic rotator cuff tears suggesting PDCP is effective as an augmentation graft in the treatment of chronic extensive rotator cuff tears, providing excellent pain relief with an improvement in active ranges of motion and strength. Synthetic materials are another option. Ciampi *et al*. 58 in a cohort study (level of evidence = 3), treated a total of 152 patients with a posterosuperior massive rotator cuff tear by open repair only, open repair together with collagen patch augmentation, or open repair together with polypropylene patch augmentation showing, the retear rate after 12 months was 41% (21/51) for the control group, 51% (25/49) for the collagen group and 17% (9/52) for the polypropylene group. They demonstrated polypropylene patch augmentation of rotator cuff repair significantly improves the 36-month outcome in terms of function, strength and retear rate. Some of these synthetic materials have low biodegradability while others have no biodegradability. Despite of excellent results, they may be present in the patient body for the entire life and may trigger foreign body reaction acutely or chronically 7,53,56. For example, Nishimoto *et al*. 59 found that using a poly-L-lactic acid as a prosthesis in a ligament defect model in rabbits, despite excellent biomechanical properties of the repaired area, the implant was not degraded and no new tissue had replaced the scaffold after 16 weeks. Also, Sato *et al*. 60 investigated several different synthetic-based artificial tendons (*e.g*. polylactic acid), the mechanical properties of which declined over 26 weeks, but were not replaced by new ligaments.

For the above reasons, most of the surgeons have tried to combine different surgical techniques and approaches for large tendon reconstruction and therefore, reconstruction of such large tissue defects is a state of art and no gold method is available 61.

We combined three-dimensional CI which was produced from the xenogenous-based collagen molecules. For more explanation, the xenogenous bovine tendons were digested to collagen molecules. The pure collagen molecules were then regenerated as collagen fibres to produce a novel three-dimensional CI. We covered such bioimplant with PDS sheath to simulate paratenon or tendon sheath. Finally, we embedded xenogenous pure bovine platelets with this construct to enhance its healing efficacy. Therefore, we tried to mimic a novel environment for the healing tissue to accelerate tendon healing. To the knowledge of the authors, neither classic grafts nor the available ECM matrices, are able to simultaneously inhibit peritendinous adhesions during tendon healing, modulate the inflammation to induce tenoinduction and tenoconduction at fibroplasia and remodelling phases of tendon healing, be gradually degraded and replaced with the new tendinous tissue, be highly incorporative with the new matrix, do not prolong the inflammation, and finally their healing behaviour be predictive. Prediction of graft behaviour after tendon reconstruction is much difficult and this is worse when a large tissue defect is aimed to be reconstructed. Actually, this is one of the main important limitations of allografts and xenografts because their healing behaviour after transplantation is unpredictable. Compared with classic grafts and surgical techniques, reconstruction of large connective tissue loss with CI-PDS-BPG is technically easier and have several surgical merits. For example, such procedure possibly decreases surgical time, does not need extensive surgical exposure, reduces pain, decreases the necessity for staged or even revision surgery, does not need to injure other tissues for tissue transferring, and has predictable outcome. Although the methodology and results of the present study, support the above statements, clinical studies should approve them. Therefore, such strategy probably reduces the surgical cost and complications. In addition, production of such bioimplant is a straight forward method with low cost.

The major merit of the present study is that we investigated the role of BPG both *in vitro* and *in vivo* using various basic to clinical methods, thus being able to suggest the BPG for tissue engineering approaches. We used clinical methods besides the basic methods to be able to better translate the results for the clinical situation. However, it should be kept in mind although animal studies have great value in medical researches, they are only an approximation 43. As stated, although auto- and allografts are currently used for reconstruction of large tendon defects, there is no well accepted method as a gold standard 1,2,5. Healing and incorporation of the grafts in these cases are completely different from each other and for this purpose, we removed comparing auto- and allografting comparisons and compared our treated tendons with more standard controls. The first control we used to compare with our treatment strategies was negative control in which we produced a defect but left it free to see the quality of spontaneous healing (the ability of the body to treat the defect). In the second control system, we compared the effectiveness of each biomaterial on tendon healing so that we compared the CI with CI-PDS and both of the CI and CI-PDS with CI-PDS-BPG. This is a well-established comparison method already being used in many of the recent investigations. In the third and final model of controlling our study, we compared the treated tendons with normal tendons which is a desired control. In fact comparing different characteristics of a healing tissue with the native or intact healthy tendon is the most accurate comparison technique because the characteristics of a normal tendon is stable and fixed between different investigations, letting to compare the results of different studies in future systematic reviews.

Although this study used large tendon defect model for evaluating the role of CI-PDS-BPG bioimplant on tendon healing, perhaps its application should not be limited for reconstruction and healing of large tendon defects. Such a novel bioimplant could be cut in any size and shape based on the site to be reconstructed. Because the CI-PDS-BPG bioimplant is fibrillar in nature and has acceptable healing properties and ability to align the newly regenerated tissue in one direction, such bioimplant could ideally be used for tendon and ligament reconstruction but could also be used for reconstruction of body wall defects and for promoting fibrosis and haemostasis in any site of the body. Because such bioimplant has fibrillar nature and have little porosity compared to the porous composites, it is not proper for reconstruction of bone and cartilage defects 6. It is necessary to test the subcutaneous biocompatibility of the CI-PDS-BPG in clinically ill patients before implantation of a large amount of scaffold in the recipient site. We showed CI-PDS-BPG had no adverse effects in rabbits while the adverse effects of such bioimplant in clinical patients is not clear.

In conclusion, we successfully developed a novel tissue-engineered bioactive graft composed of BPG, CI and PDS. The BPG significantly improved scaffold cytocompatibility *in vitro* and scaffold biocompatibility and biodegradability *in vivo*. *In vitro*, the BPG had superior activity than the PRP solutions and significantly released more growth factors. *In vivo*, the platelets directly affected tendon healing, modelling and remodelling by increasing the inflammation at short-term, accelerating the transition of inflammation to fibroplasia, enhancing the fibroplasia, and modulating the remodelling phase of tendon healing. This is the first report of the effectiveness of BPG on rabbit tendon healing. This study showed that the BPG is an accessible, reliable, cost-effective and powerful healing promotive source of the platelets and growth factors and could be considered as an alternative to autogenous and allogenous forms of platelets. The CI-PDS-BPG could be a valuable graft option, having none of the limitations of classic grafts, in managing clinically relevant tendon injuries. However, more confirmatory studies are needed before clinical application.

## Supporting Information

**Table S1** Clinical scoring criteria. **Table S2** Ultrasonographical scoring criteria. **Table S3** Gross morphological scoring criteria. **Table S4** Histological scoring criteria. **Table S5** Ultrastructural scoring criteria.
