# Supplementary material for: Synthesis, development, characterization and effectiveness of bovine pure platelet gel-collagen-polydioxanone bioactive graft on tendon healing
Source: J Cell Mol Med. 2015 Feb 20;19(6):1308–32. doi: 10.1111/jcmm.12511 (PMC4459846; doi:10.1111/jcmm.12511)
Supplement: Supplementary file 1 [file jcmm0019-1308-sd1.doc]

**Table S1: Clinical scoring criteria**

| **The tarsal flexion degree of the injured leg compared to the normal leg, both in the cage and on the floor** | | | | | | | |
| --- | --- | --- | --- | --- | --- | --- | --- |
| **Score** | **Between legs** | | | | **Estimation of the degree** | | **Condition** |
| **0** | - Equal | | | | - 75-90 | | Normal |
| **1** | - Non equal | | | | - 50-74 | | Mild |
| **2** | - Non equal | | | | - 30-50 | | Moderate |
| **3** | - Non equal | | | | - >30 | | Severe |
| **4** | - Non equal | | | | - <15 | | Extensively severe |
| **Weight distribution of each animal on the hind limbs, in the cage and on the floor** | | | | | | | |
|  | **Weight distribution between limbs** | **Weight distribution between hind legs** | | **The most weight bearing legs** | | **The injured left hind leg condition** |  |
| **0** | - Mostly hind limb | - Equal | | - Both hind limbs | | - Weight bearing | Normal |
| **1** | - Mostly hind limb | - Not equal | | - Right hind limb | | - Weight bearing | Mild |
| **2** | - Mostly forelimbs | - Not equal | | - Both forelimbs & right normal hind limb | | - Weight bearing | Moderate |
| **3** | - Not Equal | - Not equal | | - Both forelimbs and right normal hind limb | | - Non weight bearing | Severe |
| **4** | - Not Equal | - Not equal | | - Non weight bearing (sternal recumbency) | | - Non weight bearing | Extremely severe |
| **Pain in palpation of the injured area and pain in complete extension of the injured leg** | | | | | | | |
| **0** | - No reaction | | | | | | Normal |
| **1** | - Occasional vocalization | | | | | | Mild |
| **2** | - Frequent vocalization | | | | | | Moderate |
| **3** | - Vociferous vocalization, withdraw limb, bites, struggles | | | | | | Severe |
| **Heel and toe position of the injured leg (left hind paw)** | | | | | | | |
|  | **Heel** | | **toe** | | | |  |
| **0** | - Up | | - Down | | | | Normal |
| **1** | - Near the floor (up) | | - Down | | | | Mild |
| **2** | - Down | | - Down | | | | Moderate |
| **3** | - Down | | - Up | | | | Severe |
| **Swelling at the injured area (left hind paw)** | | | | | | | |
| **0** | Is not tender, warm and bowed | | | | | | Normal |
| **1** | Slightly warm and bowed, color is not changed | | | | | | Mild |
| **2** | Tenderness, bowed and completely warm. Color in not changed | | | | | | Moderate |
| **3** | Obvious tenderness, bowed and warm. Color changed. | | | | | | Severe |

**Table S2: Ultrasonographical scoring criteria**

| **Score** | 1. **Echogenicity** | 1. **Hyper echogenic area / hypo echogenic area of the tendons (homogeneity)** | 1. **Transverse movement of the tendon (Index for peritendinous adhesion)** |
| --- | --- | --- | --- |
| **0** | - Normal echogenicity | - Smooth (homogenous) echogenicity | - Free to move |
| **1** | - Slightly hyper-echoic | - Non-smooth (heterogeneous) echogenicity (mild) | - Movable in one direction (left or right) |
| **2** | - Hyper-echoic | - Non-smooth echogenicity (moderate) | - Movable in one direction with force (left or right) |
| **3** | - Hypo-echoic | - Amputated view or non-smooth echogenicity (severe) | - Fixed or non-movable |
|  | 1. **Diameter of the injured tendon / intact contralateral (regeneration volume)** | 1. **Diameter of the peritendinous low echogenic area / diameter of the high echogenic tendon (intensity of the peritendinous adhesion)** | 1. **Diameter of the proximal part of the tendon / diameter of the distal part of the tendon (regenerative proportion)** |
| **0** | - 100% | - Less than 10% | - 110-140% |
| **1** | - 90% | - 11-25% | - 91-109% |
| **2** | - 101-110% | - 26-50% | - 75-90% |
| **3** | - 75%-100% | - 50-100% | - 50-74% |
| **4** | - 50%-74% | - 100-150% | - 25-49% |
| **5** | - 0-50% | - 151% and more | - Less than 25% |

Table S3: Gross morphological scoring criteria

|  | | | |
| --- | --- | --- | --- |
| **Score** | 1. **Peritendinous adhesion** | 1. **Hyperemia** | **Status** |
| **0** | - No adhesion | - No hyperemia, shiny glistening surface appearance | Normal |
| **1** | - Tendon was easily detached from the surrounding tissues by blunt dissection | - Only in the paratenon | Mild |
| **2** | - For detachment from the surrounding tissues, tendon needed little sharp dissection | - It was extended to the tendon proper but it was not severe in nature | Moderate |
| **3** | - For detachment from the surrounding tissues, tendon needed completely sharp dissection | - It was extensively extended to the tendon proper and made its appearance more pink and dark | Severe |
| **Score** | 1. **General appearance** | **4) Muscle Atrophy** | **Status** |
| **0** | - Tendon is a unit structure (the tendinous tissue is organized as a separate tissue and could be differentiated from the surrounding structure) and is continued between the gastrocnemius muscle and calcaneal tuberosity with the same diameter and homogeneity | - The transvers diameter of the largest bulk of the muscle is more than or equal to 350% of the transvers diameter of the largest part of the Achilles tendon at its mid part. | Normal |
| **1** | - Same as above but the diameter of the injured area is larger than the proximal and distal parts of the tendon. The tendon generally is a unit structure. | - The transvers diameter of the largest bulk of the muscle is more than or equal to 250% of the transvers diameter of the largest part of the Achilles tendon at its mid part. | Mild |
| **2** | - Same as above but the diameter of the injured area is lower than the proximal and distal parts of the tendon. The tendon generally is a unit structure. | - The transvers diameter of the largest bulk of the muscle is more than or equal to 200% of the transvers diameter of the largest part of the Achilles tendon at its mid part. | Fairly moderate |
| **3** | - The injured area of the tendon is not a unit structure but the proximal and the distal parts of the tendon are a unit structure. | - The transvers diameter of the largest bulk of the muscle is more than or equal to 150% of the transvers diameter of the largest part of the Achilles tendon at its mid part. | Moderate |
| **4** | - The whole of the tendon is not a unit structure. No structure similar to tendon is seen between the gastrocnemius muscle and calcaneal tuberosity and the posterior tibialis tendon is seen. In normal condition Achilles tendon covers the posterior tibialis muscle but in this condition due to the lysis of the Achilles tendon the posterior tibialis muscle could be seen. | - The transvers diameter of the largest bulk of the muscle is more than or equal to 10% of the transvers diameter of the largest part of the Achilles tendon at its mid part. | Severe |
| **Score** | **5) Muscle fibrosis** | | **Status** |
| **0** | - No fibrosis is seen in the gastrocnemius muscle and the tendinous portion of the Achilles is the only connective tissue that covered the muscle | | Normal |
| **1** | - Mild fibrosis is seen in the muscle but more than 75% of the muscle has red color and had gross appearance similar to the muscle | | Mild |
| **2** | - Between 50 to 74% of the muscle has the characteristics of the muscle but the fibrosis is significant | | Moderate |
| **3** | - More than 50% of the muscle shows fibrosis and the fibrous tissue filled the spaces between muscle fibers. | | Severe |
| **4** | - No muscular characteristic could be seen in the gastrocnemius muscle because all of the muscle was substituted by fibrous tissue | | Extremely severe |

Table S4: Histological scoring criteria

| **Histopathologic analysis** | | | |
| --- | --- | --- | --- |
| **Score** | **1) Alignment** | **2) Perivascular edema** | **Status** |
| **0** | - Collagen fibers were longitudinally oriented in only one direction and the tenoblasts and tenocytes were laid longitudinally along their orientation | - No edema | Normal |
| **1** | - Collagen fibers were longitudinally oriented in one direction pattern but there were few areas of unorganized collagen fibers in the field | - Presence of edema just around small vessels | Mild |
| **2** | - Collagen fibers were not longitudinally oriented and the irregular orientation pattern was predominant | - Presence of edema around small and medium sized vessels | Moderate |
| **3** | - There was no obvious pattern and the collagen fibers were disorganized | - Presence of edema around all types of vessels | Severe |
| **Score** | **3) Tissue Maturity** | | **Status** |
|  | **A) the appearance of the collagen fibers** | **B) cellular populations** |  |
| **0** | - More than 75% collagen fibers are dense and they have large size | - More than 75% are fibrocytes | Normal or near normal |
| **1** | - More than 50% of the collagen fibers are dense and they are of large size | - More than 50% are fibrocytes | Highly mature |
| **2** | - More than 25% of the collagen fibers are dense and they are medium sized | - More than 25% are fibrocytes | Moderately mature |
| **3** | - The collagen fibers are not dense but they are medium sized | - More than 75% are fibroblasts | Immature |
| **4** | - The collagen fibers are not dense and they are of small sized | - The inflammatory cells are predominant | Highly immature |
| **Score** | **4) Crimp pattern** | **5) Vascularity (at remodeling stage)** | **Status** |
| **0** | - More than 75% of the collagen fibers in the light microscopic field are wavy | - No vascular structures are visible in the tissue sections. | Normal |
| **1** | - 50%-75% of the collagen fibers in the light microscopic field are wavy | - Less than 10% of the tissue density belongs to vessels. | Optimum (remodeled) |
| **2** | - 25%-50% of the collagen fibers in the light microscopic field are wavy | - Less than 25% of the tissue density belongs to vessels | Early remodeling  (fairly acceptable) |
| **3** | - Less than 25% of the collagen fibers in the light microscopic field are wavy | - Less than 50% of the tissue density belongs to vessels | Fibroplasia (bad) |
| **4** | - No crimp pattern is seen | - Less than 75% of the tissue density belongs to vessels | Early fibroplasia or degenerative changes (extremely bad) |
| **5** |  | - More than 75% of the tissue density belongs to vessels | Healing is not in progress and the newly regenerated tissue is only vascularized. |

**Table S5: Ultrastructural scoring criteria**

| **1) Alignment** | | | | |
| --- | --- | --- | --- | --- |
| **Score** | **Status** | **Directions of the collagen fibrils** | | **Direction of the fibroblasts and fibrocytes** |
| **0** | Near normal | - Most of them are aligned in one directions | | - Most of them are laid in a direction of collagen fibrils |
| **1** | Highly aligned | - More than ¾ of the collagen fibrils are aligned in one directions | | - More than ¾ of the cells are laid in a direction of collagen fibrils |
| **2** | Moderately aligned | - More than ½ of the collagen fibrils are aligned in one directions | | - More than ½ of the cells are laid in a direction of collagen fibrils |
| **3** | Fairly aligned | - More than ¼ of the collagen fibrils are aligned in one directions | | - More than ¼ of the cells are laid in a direction of collagen fibrils |
| **4** | Amorphous | - Collagen fibrils are not aligned in one direction. | | - Most of the cells are not laid in the direction of the collagen fibrils |
|  | **2) Maturity of the collagen fibrils** | | | |
| **Score** | **Status** | **Description** | | |
| **0** | Normal | - Collagen fibrils are distributed in multimodal pattern. Minimum of five different category of fibril’s diameter (extremely small (0-64nm), Small (65-102nm), medium (103-153 nm), large (154-256nm), extremely large (257-307nm)) is seen at ultra-micrographs. | | |
| **1** | Highly matured | - Collagen fibrils are distributed in multimodal pattern. Four different category of fibril’s diameter (extremely small (0-64nm), Small (65-102nm), medium (103-153nm), large (154-256nm)) is seen at ultra-micrographs. | | |
| **2** | Matured | - Collagen fibrils are distributed in multimodal pattern. Three different category of fibril’s diameter (extremely small (0-64nm), Small (65-102), medium (103-153nm)) is seen at ultra-micrographs. | | |
| **3** | Immature | - Collagen fibrils are distributed in bimodal pattern. Two different category of fibril’s diameter (extremely small (0-64nm), Small (65-102nm)) is seen at ultra-micrographs. | | |
| **4** | Highly immature | - Collagen fibrils are distributed in unimodal pattern. Only one category of fibril’s diameter (extremely small (0-64nm) is seen at ultra-micrographs. | | |
| **Score** | **3) Crimp pattern** | |  |  |
| **0** | - More than 75% of the collagen fibers in the field are wavy | |  |  |
| **1** | - 50%-75% of the collagen fibers in the field are wavy | |  |  |
| **2** | - 25%-50% of the collagen fibers in the field are wavy | |  |  |
| **3** | - Less than 25% of the collagen fibers in the field are wavy | |  |  |
| **4** | - No crimp pattern is seen | |  |  |
